# Gold-catalyzed tandem reactions of amide–aldehyde–alkyne coupling and cyclization-synthesis of 2,4,5-trisubstituted oxazoles†
†Electronic supplementary information (ESI) available: Experimental procedures and data for new compounds. See DOI: 10.1039/c5sc02933c


**DOI:** 10.1039/c5sc02933c

**Published:** 2015-10-06

**Authors:** Pierre Querard, Simon A. Girard, Nick Uhlig, Chao-Jun Li

**Affiliations:** a Department of Chemistry , FQRNT Center for Green Chemistry and Catalysis , McGill University , 801 Sherbrooke Street West , Montreal , Quebec H3A 0B8 , Canada . Email: cj.li@mcgill.ca

## Abstract

We report the first cationic gold(i)-catalyzed one-pot reaction of amide, alkyne and aldehyde followed by cyclization, to successfully access highly substituted oxazoles derivatives in good yields. A single catalyst allows the occurring of this multi-step reaction atom- and step-economically, with water as the only theoretical side product.

## Abstract 2

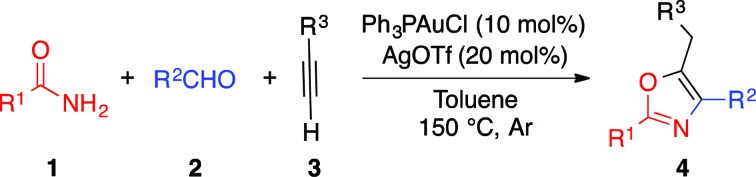
We report the first cationic gold(i)-catalyzed one-pot reaction of amide, aldehyde and alkyne followed by cyclization, to successfully access highly substituted oxazoles derivatives in good yields.

## Introduction

**Fig. 1:**
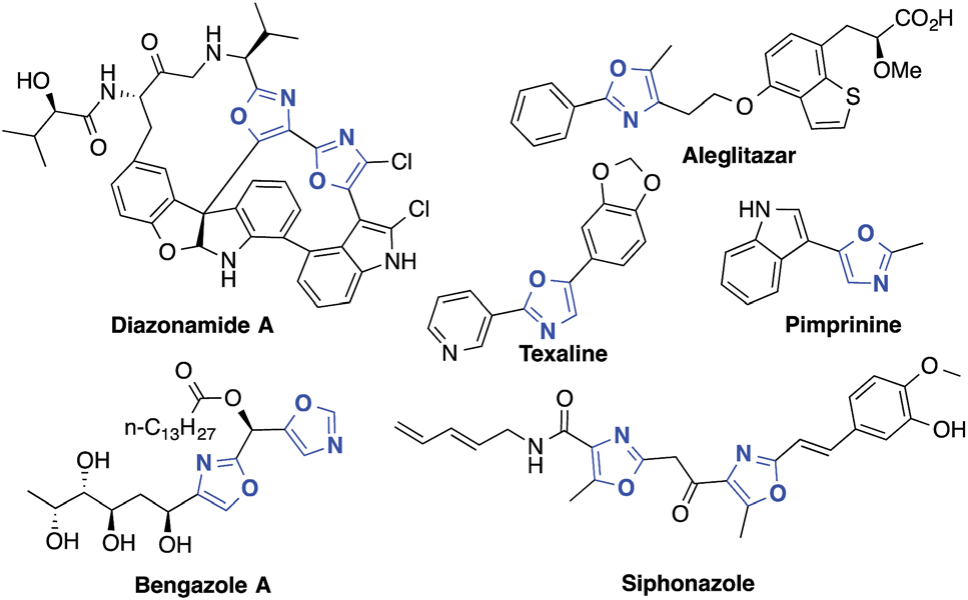
Examples of bioactive molecules and natural products containing oxazole moiety.

Oxazoles are important heterocyclic motifs present in a wide range of bioactive molecules,^1^ natural products,^2^ advanced materials,^3^ and ligand frameworks^4^ (Fig. 1). They exhibit highly variable properties and their structures are extremely diverse. As such, efficient synthetic methods accessing highly functionalized oxazoles are of great interest, yet remain challenging. Functionalization of pre-existing oxazole skeletons is one important strategy to access highly functionalized derivatives, but regioselectivity issues can limit such methods.^5^ More general synthetic pathways such as the Robinson–Gabriel^6^ and the van Leusen synthesis^7^ exploit a divergent strategy, consisting in the synthesis of acyclic oxazole precursors followed by a cyclisation.^8^ From an atom-economy perspective, such intramolecular cyclizations from acyclic precursors represent an attractive strategy for the preparation of substituted oxazoles. In the past decades, various transition metals have been reported to catalyze the cyclization of acetylenic precursors. Among these different methods, some use strong Brönsted acids or Lewis acid reagents which restrict the functional group tolerance.^9^ Thus, it is desirable to develop a simple approach to synthesize a broad variety of useful derivatives bearing diverse functionalities. Herein, we report a novel strategy for the multicomponent, one-pot synthesis of highly substituted oxazoles from simple amides, aldehydes and alkynes.

**Scheme 1:**
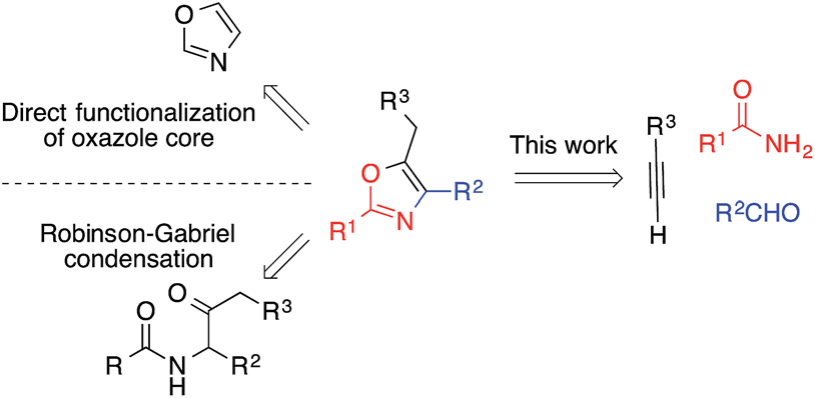
Designed strategy of one-pot gold-catalyzed A^3^/cyclization reaction.

By furnishing complex products from simple building blocks in a minimum number of steps, multicomponent reactions represent efficient and rapid alternatives to traditional stepwise syntheses.^10^ One such reaction that has proven highly versatile and useful is the aldehyde–alkyne–amine coupling (A^3^-coupling) for the formation of propargylamines.^11^ Since its discovery,^12^ the multicomponent A^3^-coupling has been extensively developed by numerous authors, and shown great promise as a tool for the synthesis of complex molecules. In particular, its amenability to tandem transformations, especially cyclization, makes it an attractive technique for the synthesis of drug-like molecules. We envisioned that oxazoles might be accessed through such a tandem A^3^-coupling–cyclization, making use of amides instead of amines (Scheme 1). However, to the best of our knowledge, the formation of propargylamides *via* the coupling of amides, aldehydes and alkynes has never been reported before.^13^ Coinage transition-metal catalysts, such as gold, have shown excellent activity for the A^3^-coupling,^14^ and have been highly effective for the cyclization of acetylenic compounds.^15^ Thus, we envisioned that a judicious choice of gold catalyst might effectively catalyze both the A^3^-coupling and the tandem cyclization steps, providing access to highly functionalized oxazoles in a single pot.^16^

## Results and discussion

Inspired by our previous work on gold-catalyzed A^3^ reactions, we began our investigation using aryl amide **1a**, alkyl aldehyde **2a** and phenylacetylene **3a** as substrates (Table 1).^17^

**Table 1:** Optimization of reaction conditions^*a*^

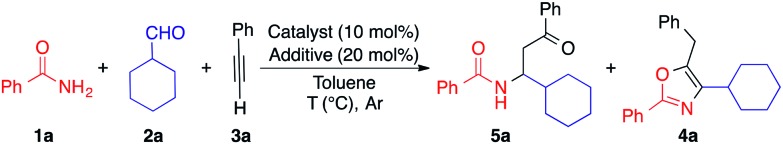					
Entry	Catalyst (10 mol%)	Additive (20 mol%)	*T* (°C)	Yield (%)	
				**5a**	**4a**
1	Ph_3_PAuCl	—	100	5	0
2	Ph_3_PAuCl	AgOTf	100	45	30
3	Ph_3_PAuCl	AgBF_4_	100	10	6
4	Ph_3_PAuCl	AgSbF_6_	100	10	7
5	Ph_3_PAuCl	AgNTf_2_	100	7	5
6^*b*^	Ph_3_PAuCl	AgOTf	100	0	0
7^*c*^	Ph_3_PAuCl	AgOTf	100	30	8
8	Ph_3_PAuCl	AgOTf	130	5	45
9	Ph_3_PAuCl	AgOTf	150	0	99 (**95**)
10	—	—	150	0	0
11	—	AgOTf	150	10	0
12	—	AgCl	150	0	0

^*a*^Reaction conditions: benzamide (0.1 mmol), cyclohexanecarboxaldehyde (0.15 mmol), phenylacetylene (0.15 mmol), toluene (0.5 mL), under argon atmosphere.

^*b*^4 Å molecular sieves were added.

^*c*^50 mol% of additive was used. All reported yields were determined by ^1^H NMR spectroscopy of the crude reaction mixture using dibromomethane as internal standard. Yields in brackets are isolated.

**Scheme 2:**
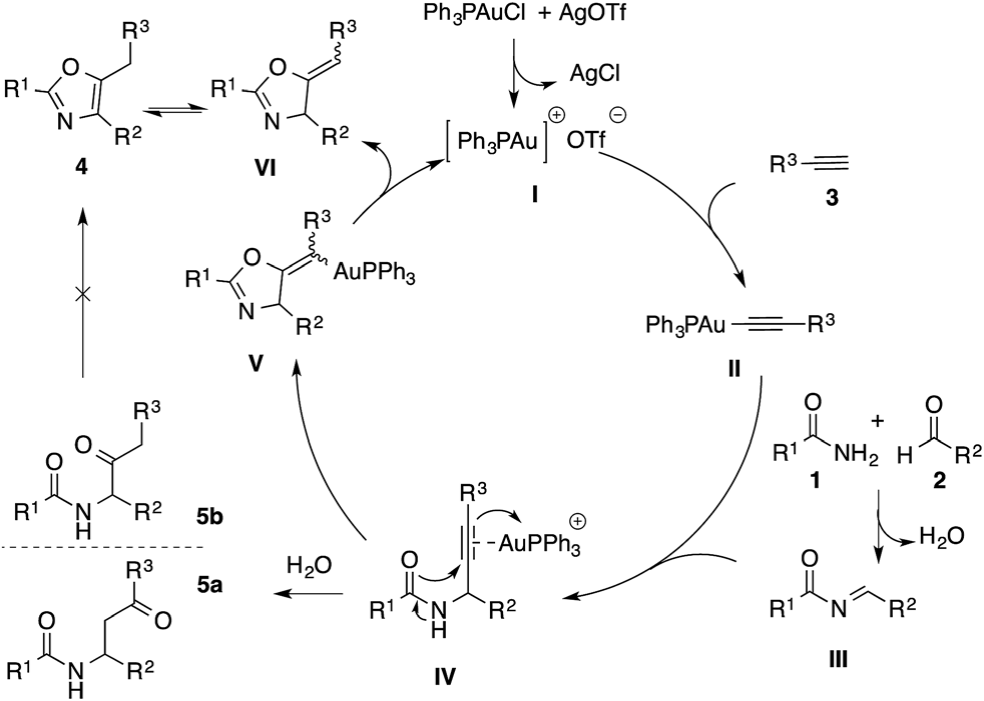
Proposed mechanism for the A^3^ coupling/cyclo-addition reaction.

While triphenylphosphinegold(i) chloride on its own did not generate any desired product, the addition of silver(i) triflate furnished product **4a** in 30% yield (entry 2). The counter-anion of silver salt dramatically influenced the yields of the reaction, with triflate giving the best result (entries 2–5). When Ph_3_PAuCl/AgOTf was used in toluene at 100 °C, a significant amount of 3-acylamidoketone **5a** was detected, as well as its regioisomer **5b** in a trace amount (<10%, see Scheme 2). To investigate the influence of water on the formation of this side-product, 4 Å molecular sieves were added (entry 6), which resulted in the complete inhibition of the desired reaction possibly due to gold poisoning from the molecular sieves.^18^ While it has been reported that a suitable acid activator (*i.e.* AgOTf) prevents the degradation of the gold catalyst,^18^ the addition of 50 mol% AgOTf was not beneficial to the reaction (entry 7). Although only a slight improvement of the reaction yield was observed at 130 °C, increasing the reaction temperature to 150 °C drastically accelerated the reaction, leading to complete conversion and excellent yield of the desired product (entries 8 and 9). In the absence of metal catalyst or additive, no desired product was observed (entry 10–12). The silver chloride formed during the catalyst preformation likewise showed no activity in the reaction (entry 12).

With the optimized conditions in hand, we investigated the reaction scope (Table 2). We were pleased to find that both aliphatic and aromatic aldehydes delivered the corresponding oxazoles in moderate to excellent yields.

**Table 2:** Amide, aldehyde, alkyne coupling – formation of 2,4,5 tri-substituted oxazoles^*a*^

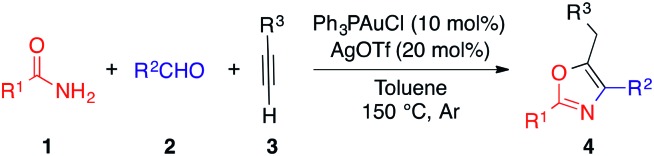
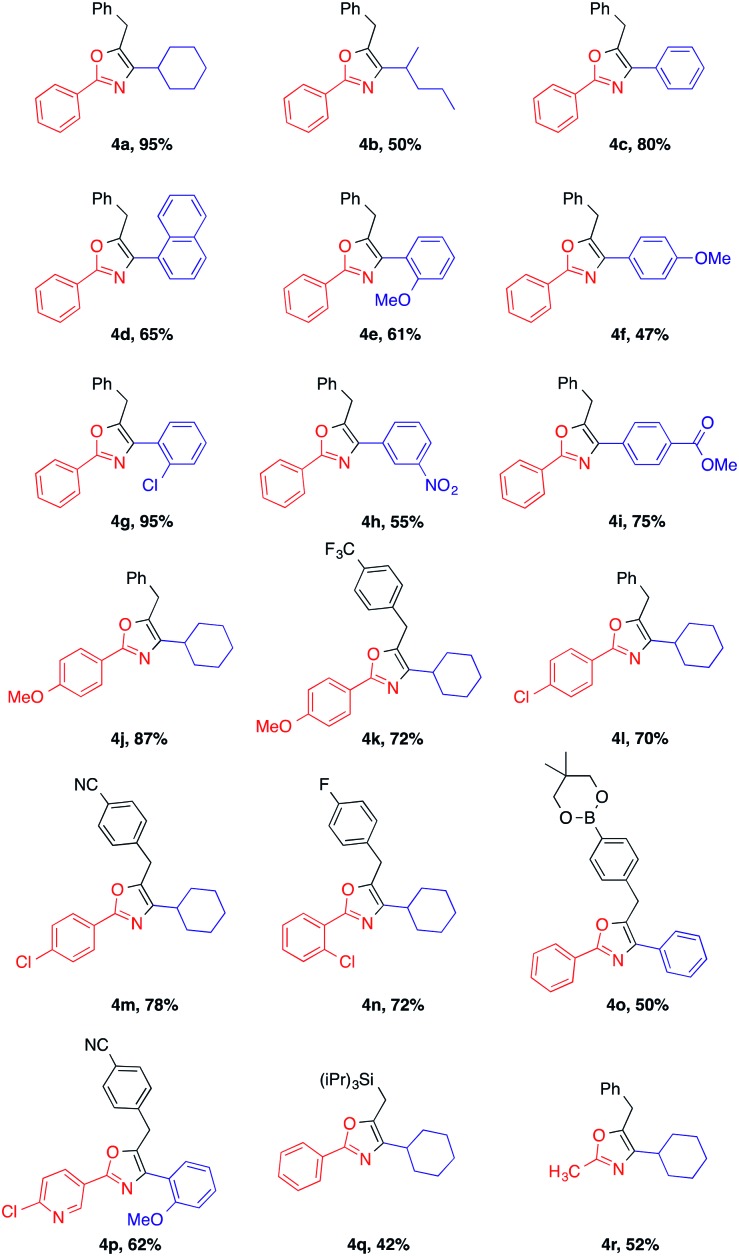

^*a*^Conditions: amides (0.2 mmol), aldehydes (0.3 mmol), alkynes (0.3 mmol), Ph_3_PAuCl (10 mol%), AgOTf (20 mol%), 0.5 mL of toluene, 6 h, under argon. Isolated yields reported.

Cyclohexanecarboxaldehyde **2a** reacted with the coupling partners to afford the substituted oxazole **4a** in a significantly better yield (95% isolated yield) than acyclic aliphatic aldehyde **2b** (**4b**). Aromatic aldehydes with various functional groups were well tolerated and the corresponding products were isolated in good to excellent yields (**4c** and **4d**). While aromatic aldehydes with both electron-withdrawing groups (EWG) and -donating groups (EDG) were well tolerated under the reaction conditions, aldehydes bearing EWGs such as chloride and ester substituents generally provide the desired product (**4g** and **4i**, respectively) in higher yields compared to the ones bearing EDGs such as –OMe (**4e** and **4f**). Aromatic amides possessing different EWGs and EDGs were also evaluated, and resulted in good reaction yields (**4j–n**). It is noteworthy that even a boronic ester was tolerated under the reaction conditions, providing a handle for further functionalization *via* Suzuki coupling (**4o**). Impressively, our method can be further extended to the substrate bearing heterocyclic compound. The reaction of 4-chloronicotinamide afforded the oxazole product **4p** in 62% yield. Fortunately, subjecting the substrate triisopropylsilyl acetylene **3q** to the standard reaction conditions could successfully afford the corresponding oxazole heterocyclic compound **4q**, albeit in a slightly lower yield. Besides, alkyl amide, such as acetamide, exposed to our reaction system produced **4r** in a moderate yield.

Our proposed mechanism to rationalize this reaction is presented in Scheme 2. The abstraction of chloride from triphenylphosphinegold chloride complex by silver salt generates the active cationic gold species **I**, which reacts with phenylacetylene to form the gold acetylide **II**.^19^ Simultaneously, the condensation reaction between amide **1** and aldehyde **2** results in the formation of imide **III**. The subsequent addition of gold acetylide **II** to imide **III** affords propargylamide **IV**. Then the coordination of cationic gold species to alkyne can further assist either the intramolecular 5-*exo*-dig cyclization (towards the formation of cyclic organogold complex **V**), or the formation of hydrated side products **5_a_** and **5_b_**.^17^ It is noteworthy that in our experiments, these hydrated side-products were produced exclusively at lower temperature. To determine the fate of these side-products, control experiments with and without gold catalyst were conducted under our optimized reaction conditions. We observed that compounds **5_a_** and **5_b_** did not lead to the formation of the corresponding oxazoles. Finally, succeeding the formation of **V**, the oxazoline intermediate **VI** is obtained *via* protodeauration, which further tautomerizes into the desired tri-substituted oxazole product **4**.

## Conclusions

In summary, we have successfully developed a highly efficient one-pot coupling method for the direct synthesis of tri-substituted oxazoles *via* an unprecedented amide, aldehyde and alkyne coupling (A^′^A^2^). Using the tool of a single cationic gold(i) catalyst in one-pot to accomplish both the A^′^A^2^ and the cycloaddition reactions, provides a novel atom-economical and practical alternative to construct important heterocyclic compounds, with water as the only side product. We further envisioned that this tandem reaction could be extended towards many other synthetically useful motifs and the expansion of the scope of simple starting material is currently undergoing in our laboratory.

## Supplementary Material

Supplementary information
