# Supplementary material for: Gold-catalyzed tandem reactions of amide–aldehyde–alkyne coupling and cyclization-synthesis of 2,4,5-trisubstituted oxazoles
Source: Chem Sci. 2015 Oct 6;6(12):7332–5. doi: 10.1039/c5sc02933c (PMC5950834; doi:10.1039/c5sc02933c)
Supplement: Supplementary file 1 [file SC-006-C5SC02933C-s001.pdf]

Electronic Supplementary Material (ESI) for Chemical Science.
This journal is © The Royal Society of Chemistry 2015

Gold-Catalyzed Tandem Reactions of Amide-Aldehyde-Alkyne Coupling and Cyclization - Synthesis of 2,4,5-Trisubstituted Oxazoles

Pierre Querard, [a] Simon A. Girard, [a] Nick Uhlig [a] and Chao-Jun Li [a,*]

Department of Chemistry, and FQRNT Center for Green Chemistry and Catalysis, McGill University, 801 Sherbrooke Street West, Montreal, Quebec H3A 0B8, Canada

cj.li@mcgill.ca

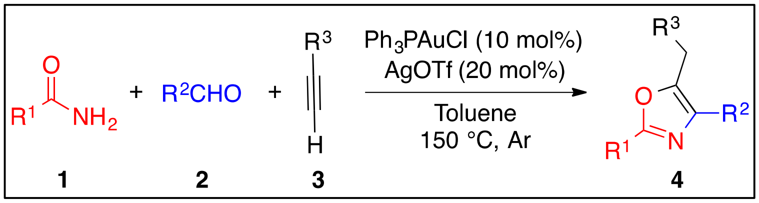

Supplementary Information

<u>**Contents**</u>

1. General Information
2. Experimental details and characterization data for all compounds
   a. **Selected reaction conditions**
   b. **Study of protected amide**
   c. **Control experiments concerning 5a (side-product)**
3. ^{1}H NMR and ^{13}C NMR spectra for new compounds

1. General Information

Solvents and reagents were purchased from Sigma-Aldrich chemical company and were used without further purification unless otherwise specified. NMR spectra were recorded on a Bruker AV500 spectrometer operating respectively at 500 MHz and 126 MHz for ^1H and ^{13}C acquisitions.
Chemical shifts are reported in ppm with a solvent resonance as an internal standard (^1H-NMR; chloroform as internal standards, indicating 7.26ppm, ^{13}C-NMR; chloroform as internal standard, indicating 77.00ppm). Data are reported as following: chemical shift, multiplicity (s = singlet, d = doublet, dd = doublet of doublets, t = triplet, q = quartet, m = multiplet, br = broad signal) and integration.
High-resolution mass spectrometry was conducted using atmospheric pressure chemical ionization (APCI) or electro-spraying ionization (ESI), and was performed by McGill University on a Thermo-Scientific Exactive Orbitrap. Protonated molecular ions (M+H)+ or sodium adducts (M+Na)+, were used for empirical formula confirmation. All preparative chromatography were performed using gradient elution (hexanes and ethyl acetate) on a Biotage Isolera™ One automated chromatography system with SNAP ultra silica gel cartridges and sample cartridges.

2 Experimental details and characterization data for all compounds

General Procedure for optimization of reactions conditions

A V-shaped 10 mL Biotage reaction vial was charged with Ph_3PAuCl (10 mol%, 5.0 mg), AgOTf (20 mol%, 5.0 mg), and the corresponding benzamide (0.1 mmol), evacuated and refilled with argon three times. Freshly distilled toluene (0.25 mL) was added followed by subsequent addition of cyclohexane carboxaldehyde (0.15 mmol) and phenylacetylene (0.15 mmol). The reaction vessel was sealed, placed in an oil bath pre-heated at 150 °C under vigorous stirring (approx. 1400 rpm) and hold for 6 hours. The mixture was cooled to room temperature, diluted with ethyl acetate, filtered through a pad of silica, and rinsed with additional ethyl acetate. The combined rinsing were concentrated and purified by column chromatography or preparative thin layer chromatography to yield the corresponding oxazoles **4**.
Dibromomethane was used as internal standard for ^1H-NMR analysis.

Selected reaction conditions

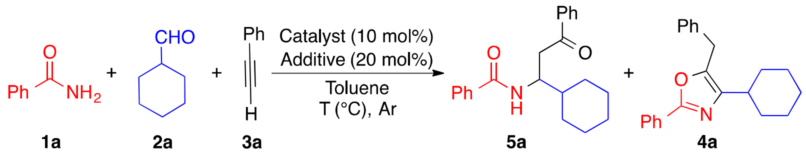

Table S1: Screening and optimization of reaction conditions[1]

Entry	Catalyst (10 mol%)	Additive (20 mol%)	T (°C)	Yield (NMR %) 5a	4a
1	$Cu(OTf)_2$	-	100	35	0
2	$Cu(OTf)_2$	PTSA (1 eq)	100	0	0
3	$Cu(OTf)_2$	KOAc (1eq)	100	10	0
4	$(Cu(OTf))_2$	-	100	10	0
5	CuCN	-	100	3	0
6	$CuCl_2$	-	100	12	0
7	CuBr	-	100	0	0
8	CuBr	$RuCl_3$	100	0	0
9	AgOTf	-	100	58	0
10	AgOTf	H_20 (1 eq)	100	40	0
11	AgCl	-	100	0	0
12	$AuCl(SMe)_2$	-	100	10	0
13	$AuBr_3$	-	100	15	0

We started the optimization based on our previous work,[2] on Copper(II) Triflate-Catalyzed Three-Component Coupling of Aldehydes, Alkynes and Carbamates. Following the optimal conditions developed previously, copper(II) triflate as the catalyst in toluene under 100°C, we were please to see the formation of **5a** in 35% yield (entry 1). In order to develop our designed reaction, we looked at the metal acetylide formation, which is a crucial step. Thus the use of organic or inorganic bases in order to help the metal acetylide formation via deprotonation of the terminal alkyne was evaluated. Unfortunately, the use of acid or base (entries 2 and 3) was not beneficial for the formation of **4a**. Copper salt were screened and evaluated in table S1, however **4a** was never detected.

Coinage transition-metal catalysts, such as gold, have shown excellent activity for the A^3-coupling, and have been highly effective for the cyclization of acetylenic compounds. Thus, we envisioned that a judicious choice of gold catalyst might effectively catalyze both the A^3-coupling and the tandem cyclization steps

Formation of metal-acetylide with silver or gold has been shown in many examples.[3] However, gold(I), gold(III) and silver metal catalysts were not efficient to conduct the formation of **4a**. Nevertheless, we were pleased to see that 60% of **5a** was produced in the presence of silver triflate (entry 9). We hypothesized that **5a** was formed via hydration by water, which was delivered during the process. Addition of 1 equivalent of water was not beneficial for the reaction, since the yield dropped to 40% (entry 10).

---

[1] All reported yields were determined by ^1H NMR spectroscopy using dibromomethane as internal standard.

[2] X.-Y. Dou, Q. Shuai, L.-N. He and C.-J. Li, *Adv. Synth. Catal.*, 2010, **352**, 2437

[3] (a) J. Bucher, T. Wurm, K. S. Nalivela, M. Rudolph, F. Rominger and A. S. K. Hashmi, *Angew. Chem. Int. Ed.*, 2014, **53**, 3854; (b) D. J. Gorin and F. D. Toste, *Nature*, 2007, **446**, 395.

Table S2: Screening and optimization of reaction conditions[4]

Entry	Catalyst (10 mol%)	Additive (20 mol%)	T (°C)	Yield (NMR %) 5a	4a
1	AgOTf	DCE	100	44	0
2	AgOTf	MeCN	100	*nd*	0
3	AgOTf	THF	100	8	0
4	AgOTf	Dioxane	100	14	0
5	AgOTf	H_2O	100	0	0
6	AgOTf	MeOH	100	*nd*	0
7	AgOTf	DMSO	100	*nd*	0

After a screening of a broad range of different solvent in term of polarity and proticity, toluene demonstrated the best properties for the formation of **5a** (entry 9). In acetonitrile, MeOH and DMSO, we were not able to determine the yield of **5a** due to signals overlap.
After extensive screening of conditions using copper and silver transition metal catalyst, without significant results, we focused our attention on gold catalysis.

Table S3: Screening and optimization of reaction conditions

Entry	Catalyst (10 mol%)	Additive (20 mol%)	T (°C)	Yield (NMR %) 5a	4a
1	AuCl	iPr.Cl	100	5	0
2	Ph_3PAuCl	AgOTf	100	45	**30**
3	Et_3PAuCl	AgOTf	100	44	14
4	iMesAuCl	AgOTf	100	38	1
5	Ph_3PAuCl	AgOTf + acetic anhydride (1 eq)	100	2	2
6	Ph_3PAuCl	AgOTf + 4A or 5A Molecular sieves	100	0	0
7	Ph_3PAuCl	AgOTf + $MgSO_4$	100	0	0
8	Ph_3PAuCl	AgOTf + Na_2SO_4	100	33	0
9	$AuCl_3$	AgOTf	100	46	0

We were extremely please to see the formation of compound **4a** when gold(I) cationic catalyst was used (entry 2). Evaluating other electronic properties of the phosphine ligands, we did not managed to improve the yield above 30% (entry 3). Other types of ligands, such as N-heterocyclic carbene, were not efficient for

[4] "nd" : not determined. NMR signals of related product could not clearly be analyzed due to overlapping signals.

the reaction (entry 4). Seeing the overall yield of the reaction reaching the 75%, we wanted to be able to control the selectivity towards the formation of **4a**. Our hypothesis was to trap the water released during the condensation via different methods. Acetic anhydride was used as a water trap, but it resulted in a decrease in yield (entry 5). Dehydrating agents (molecular sieves, $MgSO_4$) have shown strong impact on the reaction yield (entries 6-7) but none of them have shown any improvement and even completely killed the reaction.

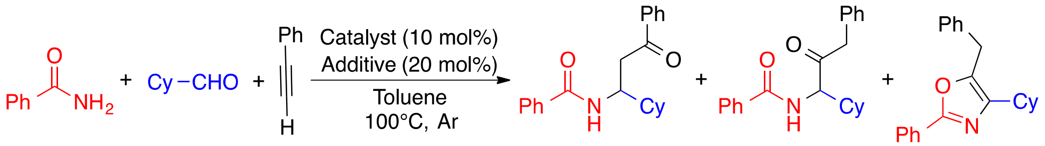

Table S4: Study of reaction time

Entry	Catalyst / Additive	Time	Yield (NMR %) 5a	5b	4a
1	Ph_3PAuCl / AgOTf	3h	18	11	7
2	Ph_3PAuCl / AgOTf	6h	45	10	30
3	Ph_3PAuCl / AgOTf	18h	43	12	26
4	Ph_3PAuCl / AgOTf	64h	50	10	25

Evaluating the reaction time of our system, no big difference in term of NMR yield was observed. Nevertheless, those results allowed us to get more information about the mechanism of the reaction. Once the hydrated side products **5a** or **5b** were formed, their transformation to the corresponding oxazole moiety was not detected after prolonged reaction time under our reaction condition suggesting that the Robinson-Gabriel pathway is unlikely.

Table S5: Study of reaction temperature.

Entry	Catalyst (10 mol%)	Additive (20 mol%)	T (°C)	Yield % (NMR) 5a	4a
1	Ph_3PAuCl	-	100	5	0
2	Ph_3PAuCl	AgOTf	100	45	30
3	Ph_3PAuCl	AgOTf	130	5	45
4	Ph_3PAuCl	AgOTf	150	0	99 (**95**)
5	-	-	150	0	0
6	-	AgOTf	150	10	0

While triphenylphosphinegold(I) chloride on its own did not generate any desired product, the addition of silver(I) triflate furnished product **4a** in 30% yield (entry 2). The counter-anion of silver salt dramatically influenced the yields of the reaction, with triflate giving the best result. When Ph_3PAuCl/AgOTf was

used in toluene at 100 °C, a significant amount of 3-acylamidoketone **5a** was detected, as well as its regioisomer **5b** in a trace amount (< 10%, see Scheme 2). Although a slight improvement of the reaction yield was observed at 130 ˚C, increasing the reaction temperature to 150 ˚C drastically accelerated the reaction, leading to complete conversion and excellent yield of the desired product (entries 3-4). In the absence of metal catalyst or additive, no desired product was observed (entry 5-6).

Study of protected amide

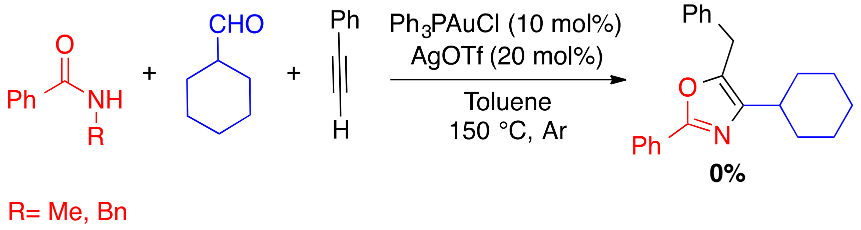

N-protected benzamide were studied in our reaction condition. However the reactions were not successful in forming the corresponding oxazoles. The starting materials were recovered.

Control experiments concerning 5a (side-product)

Scheme S1:

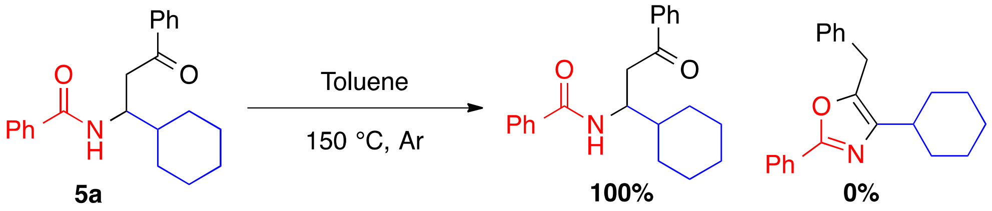

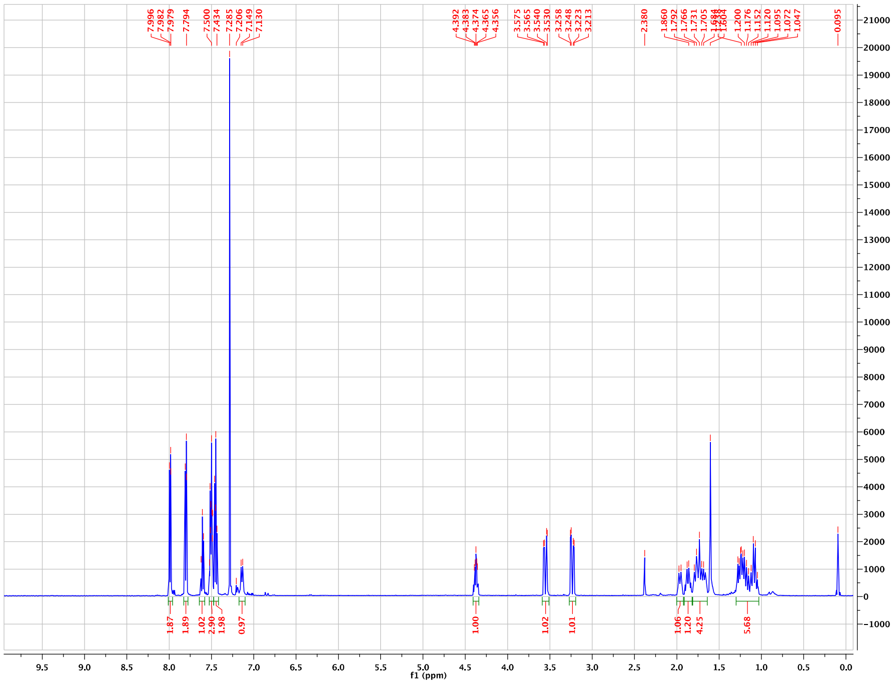

Scheme S2:

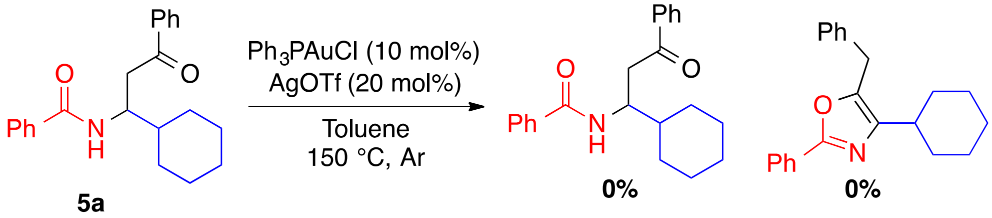

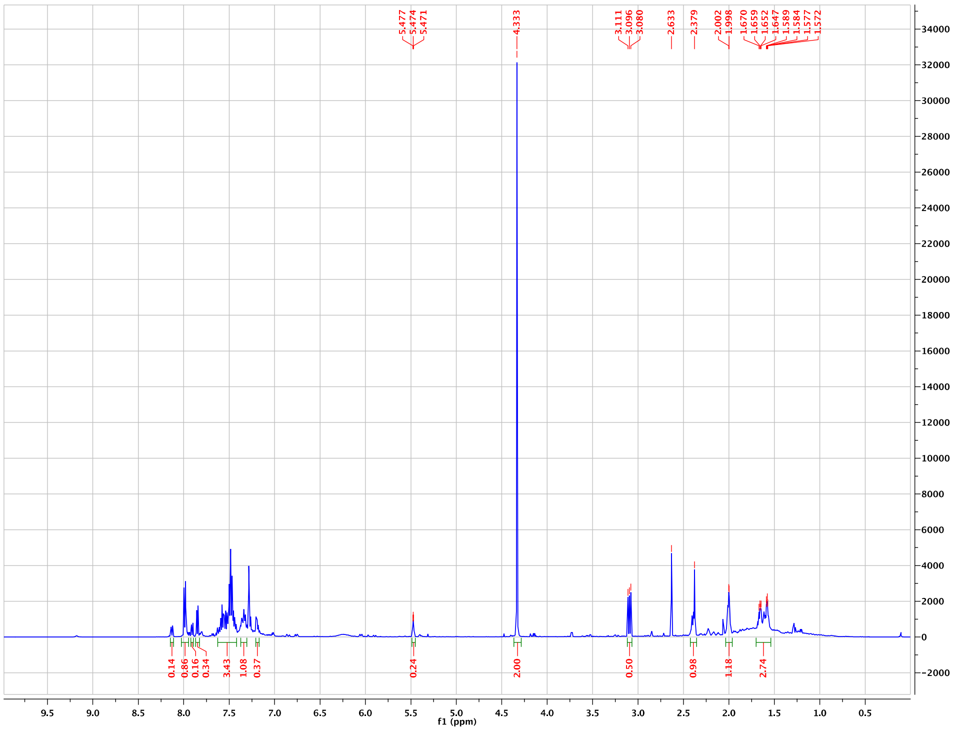

5.477
5.474
5.471
4.333
3.111
3.096
3.080
2.633
2.379
2.002
1.998
1.670
1.659
1.652
1.647
1.589
1.584
1.577
1.572
0.14
0.86
0.16
0.34
3.43
1.08
0.37
0.24
2.00
0.50
0.98
1.18
2.74
f1 (ppm)

3 ^{1}H NMR and ^{13}C NMR spectra for new compounds

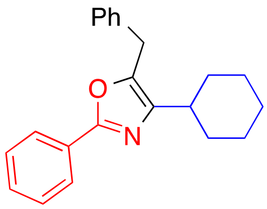

4a

Use the general procedure described above, compound **4a** was obtained from benzamide (0.2 mmol, 24.2 mg), cyclohexane carboxaldehyde (0.3 mmol, 36.3 uL) and phenylacetylene (0.3 mmol, 34 uL) as a white solid (62.7 mg) in 95% yield.
^{1}H NMR ($CDCl_3$, 500 MHz): δ = 8.00-7.98 (m, 2H), 7.43-7.39 (m, 3H), 7.33-7.32 (m, 2H), 7.27-7.25 (m, 3H), 4.08 (s, 2H), 2.61-2.55 (tt, J = 11.7, 3.5 Hz, 1H), 1.87−1.80 (m, 4H), 1.74−1.69 (m, 3H), 1.38−1.34 (m, 3H).
^{13}C NMR ($CDCl_3$, 126 MHz): δ = 159.75, 143.95, 142.13, 138.00, 129.62, 128.60, 128.52, 128.32, 128.02, 126.56, 126.12, 35.67, 32.41, 31.14, 26.55, 25.87.
HRMS (ESI) m/z: [M + H]+ calculated for $C_{22}H_{24}NO$ 318.1858, found 318.18585.
Rf (hexane/EtOAc 4:1): 0.5.

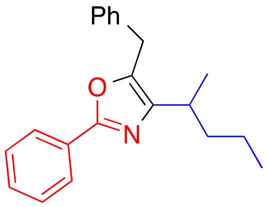

4b

Use the general procedure described above, compound **4b** was obtained from benzamide (0.2 mmol, 24.2 mg), 2-methylpentanal (0.3 mmol, 37 uL) and phenylacetylene (0.3 mmol, 34 uL) as a white solid (30.5 mg) in 50% yield.
^{1}H NMR ($CDCl_3$, 500 MHz): δ = 8.01-7.99 (m, 2H), 7.42-7.39 (m, 3H), 7.35-7.32 (m, 2H), 7.27-7.25 (m, 3H), 4.05 (s, 2H), 2.73-2.80 (sext, J = 6.8 Hz, 1H), 1.76−1.70 (m, 1H), 1.61−1.54 (m, 1H), 1.29-1.28 (d, J = 6.9 Hz, 3H), 1.28−1.24 (m, 1H), 0.99-0.92 (m, 1H), 0.87-0.90 (t, J = 7.4 Hz, 3H).
^{13}C NMR ($CDCl_3$, 126 MHz): δ = 159.89, 144.46, 141.62, 137.95, 129.63, 128.52, 128.48, 128.36, 128.06, 126.56, 126.15, 38.44, 31.02, 30.60, 20.87, 20.58, 14.07.
HRMS (ESI) m/z: [M + H]+ calculated for $C_{21}H_{24}NO$ 306.1858, found 306.18542.
Rf (hexane/EtOAc 4:1): 0.5.

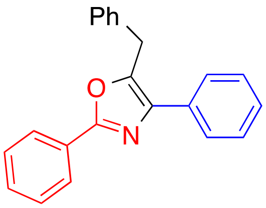

4c

Use the general procedure described above, compound **4c** was obtained from benzamide (0.2 mmol, 24.2 mg), benzaldehyde (0.3 mmol, 32 uL) and phenylacetylene (0.3 mmol, 34 uL) as a white solid (49.7 mg) in 80% yield.
^{1}H NMR (CDCl$_3$, 500 MHz): δ = 8.11-8.10 (m, 2H), 7.78-7.76 (m, 2H), 7.49-7.44 (m, 5H), 7.38-7.32 (m, 5H), 7.30-7.28 (m, 1H), 4.34 (s, 2H).
^{13}C NMR (CDCl$_3$, 126 MHz): δ = 160.18, 145.62, 137.26, 137.23, 132.06, 130.15, 128.80, 128.69, 128.29, 127.72, 127.54, 127.05, 126.81, 126.34, 31.96.
HRMS (ESI) m/z: [M + H]+ calculated for $C_{22}H_{18}NO$ 312.1388, found 312.13873.
Rf (hexane/EtOAc 4:1): 0.4.

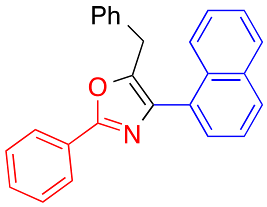

4d

Use the general procedure described above, compound **4d** was obtained from benzamide (0.2 mmol, 24.2 mg), 1-naphthaldehyde (0.3 mmol, 40.8 uL) and phenylacetylene (0.3 mmol, 34 uL) as a white solid (46.9 mg) in 65% yield.
^{1}H NMR (CDCl$_3$, 500 MHz): δ = 8.16-8.12 (m, 3H), 7.94-7.92 (m, 2H), 7.57-7.51 (m, 5H), 7.50-7.46 (m, 3H), 7.33-7.30 (m, 2H), 7.26-7.24 (m, 3H), 4.11 (s, 2H).
^{13}C NMR (CDCl$_3$, 126 MHz): δ = 160.30, 147.57, 137.37, 136.66, 133.95, 132.25, 130.17, 129.17, 129.00, 128.17, 128.64, 128.49, 128.27, 127.86, 127.63, 126.69, 126.41, 126.36, 126.07, 126.03, 125.21, 31.47.
HRMS (ESI) m/z: [M + H]+ calculated for $C_{26}H_{20}NO$ 362.1545, found 362.15439.
Rf (hexane/EtOAc 4:1): 0.6.

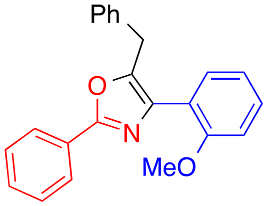

4e

Use the general procedure described above, compound **4e** was obtained from benzamide (0.2 mmol, 24.2 mg), 2-methoxybenzaldehyde (0.3 mmol, 36.3 uL) and phenylacetylene (0.3 mmol, 34 uL) as a white solid (41.5 mg) in 61% yield.
^{1}H NMR (CDCl$_3$, 500 MHz): δ = 8.09-8.07 (m, 2H), 7.63-7.61 (m, 1H), 7.46-7.43 (m, 3H), 7.39-7.36 (m, 1H), 7.33-7.27 (m, 4H), 7.27-7.24 (m, 1H), 4.15 (s, 2H), 3.75 (s, 3H).
^{13}C NMR (CDCl$_3$, 126 MHz): δ = 160.26, 156.72, 147.35, 137.87, 133.91, 131.31, 129.78, 129.58, 128.60, 128.51, 128.41, 127.64, 126.40, 126.30, 121.06, 120.84, 111.08, 55.32, 32.15.
HRMS (ESI) m/z: [M + H]+ calculated for $C_{23}H_{20}NO_2$ 342.1494, found 342.14942.

Rf (hexane/EtOAc 4:1): 0.6.

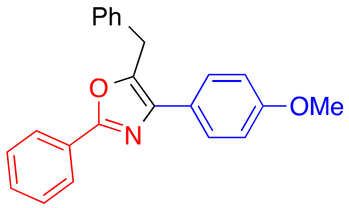

4f

Use the general procedure described above, compound **4f** was obtained from benzamide (0.2 mmol, 24.2 mg), 4-methoxybenzaldehyde (0.3 mmol, 36.6 uL) and phenylacetylene (0.3 mmol, 34 uL) as a white solid (32.0 mg) in 47% yield.
^{1}H NMR ($CDCl_3$, 500 MHz): δ = 8.10-8.08 (m, 2H), 7.70-7.67 (m, 2H), 7.48-7.43 (m, 3H), 7.37-7.28 (m, 4H), 7.00-6.97 (m, 2H), 4.31 (s, 2H), 3.86 (s, 3H).
^{13}C NMR ($CDCl_3$, 126 MHz): δ = 160.00, 159.23, 144.70, 137.40, 137.05, 130.04, 128.77, 128.65, 128.31, 128.26, 127.63, 126.75, 126.26, 124.69, 114.13, 55.32, 31.91.
HRMS (ESI) m/z: [M + H]+ calculated for $C_{23}H_{20}NO_2$ 342.1494, found 342.14943.
Rf (hexane/EtOAc 4:1): 0.6.

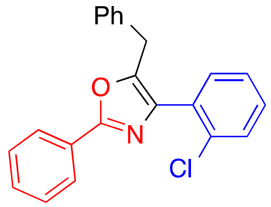

4g

Use the general procedure described above, compound **4g** was obtained from benzamide (0.2 mmol, 24.2 mg), 2-chlorobenzaldehyde (0.3 mmol, 33.6 uL) and phenylacetylene (0.3 mmol, 34 uL) as a white solid (65.7 mg) in 95% yield.
^{1}H NMR ($CDCl_3$, 500 MHz): δ = 8.08-8.06 (m, 2H), 7.52-7.49 (m, 2H), 7.46-7.45 (m, 3H), 7.36-7.34 (m, 2H), 7.32-7.29 (m, 2H), 7.25-7.24 (m, 3H), 4.11 (s, 2H).
^{13}C NMR ($CDCl_3$, 126 MHz): δ = 160.31, 147.51, 136.98, 135.12, 133.96, 132.04, 131.07, 130.21, 129.94, 129.79, 128.69, 128.57, 128.56, 127.44, 126.81, 126.70, 126.31, 55.32, 31.90.
HRMS (ESI) m/z: [M + H]+ calculated for $C_{22}H_{17}ClNO$ 346.0999, found 346.09961.
Rf (hexane/EtOAc 4:1): 0.6.

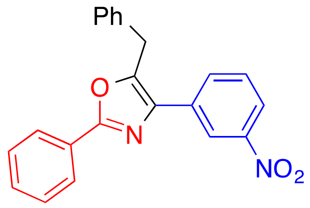

4h

Use the general procedure described above, compound **4h** was obtained from benzamide (0.2 mmol, 24.2 mg), 3-nitrobenzaldehyde (0.3 mmol, 45.3 mg), and phenylacetylene (0.3 mmol, 34 uL) as a white solid (65.7 mg) in 55% yield.
^{1}H NMR ($CDCl_3$, 500 MHz): δ = 8.64 (s, 1H), 8.18-8.19 (m, 1H), 8.12-8.08 (m, 3H), 7.62-7.58 (m, 1H), 7.50-7.49 (m, 3H), 7.39-7.33 (m, 4H), 7.31-7.29 (m, 1H), 4.38 (s, 2H).
^{13}C NMR ($CDCl_3$, 126 MHz): δ = 160.51, 148.57, 147.12, 136.28, 135.04, 133.86, 132.55, 130.56, 129.62, 128.99, 128.82, 128.63, 128.30, 127.14, 126.41, 122.25, 121,76, 32.19.
HRMS (ESI) m/z: [M + H]+ calculated for $C_{22}H_{17}N_2O_3$ 357.1239, found 357.12347.
Rf (hexane/EtOAc 4:1): 0.6.

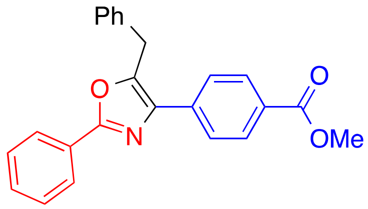

4i

Use the general procedure described above, compound **4i** was obtained from benzamide (0.2 mmol, 24.2 mg), methyl 4-formylbenzoate (0.3 mmol, 49.2 mg) and phenylacetylene (0.3 mmol, 34 uL) as a white solid (83.1 mg) in 75% yield.
^{1}H NMR ($CDCl_3$, 500 MHz): δ = 8.13-8.09 (m, 4H), 7.86-7.84 (m, 2H), 7.49-7.47 (m, 3H), 7.36-7.35 (m, 2H), 7.32-7.29 (m, 3H), 4.37 (s, 2H), 3.95 (s, 3H).
^{13}C NMR ($CDCl_3$, 126 MHz): δ = 166.86, 160.41, 146.91, 136.72, 136.54, 136.34, 130.38, 130.00, 129.10, 128.89, 128.75, 128.24, 127.27, 126.98, 126.71, 126.39, 52.13, 32.17.
HRMS (ESI) m/z: [M + H]+ calculated for $C_{24}H_{20}NO_3$ 370.1443, found 370.14462.
Rf (hexane/EtOAc 4:1): 0.5.

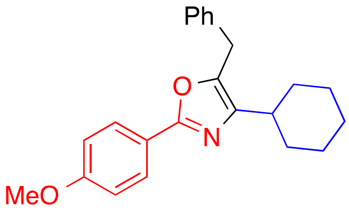

4j

Use the general procedure described above, compound **4j** was obtained from 4-methoxybenzamide (0.2 mmol, 30.24 mg), cyclohexane carboxaldehyde (0.3 mmol, 36.3 uL) and phenylacetylene (0.3 mmol, 34 uL) as a white solid (90.6 mg) in 87% yield.
^{1}H NMR ($CDCl_3$, 500 MHz): δ = 7.93-7.91 (m, 2H), 7.35-7.32 (m, 2H), 7.28-7.25 (m, 3H), 6.94-6.92 (m, 2H), 4.06 (s, 2H), 3.85 (s, 3H), 2.59-2.54 (tt, J = 11.7, 3.5 Hz, 1H), 1.86−1.80 (m, 4H), 1.73−1.67 (m, 3H), 1.38−1.28 (m, 3H).

^{13}C NMR (CDCl$_3$, 126 MHz): δ = 160.81, 159.83, 143.29, 141.82, 138.16, 128.58, 128.31, 127.75, 126.51, 120.96, 113.92, 55.32, 35.70, 32.42, 31.12, 26.57, 25.88.
HRMS (ESI) m/z: [M + H]+ calculated for $C_{23}H_{26}NO_2$ 348.1964, found 348.19570.
Rf (hexane/EtOAc 4:1): 0.5.

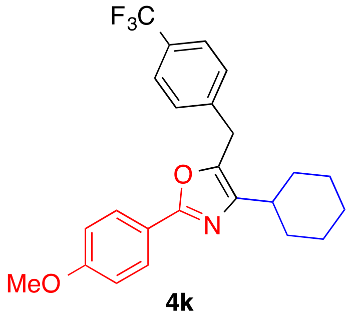

4k

Use the general procedure described above, compound **4k** was obtained from 4-methoxybenzamide (0.2 mmol, 30.24 mg), cyclohexane carboxaldehyde (0.3 mmol, 36.3 uL) and 1-ethynyl-4-(trifluoromethyl) benzene (0.3 mmol, 32.6 uL) as a white solid (59.8 mg) in 72% yield.
^{1}H NMR (CDCl$_3$, 500 MHz): δ = 7.92-7.90 (m, 2H), 7.59-7.58 (m, 2H), 7.37-7.35 (m, 2H), 6.94-6.92 (m, 2H), 4.11 (s, 2H), 3.85 (s, 3H), 2.58-2.53 (tt, J = 11.7, 3.5 Hz, 1H), 1.87−1.79 (m, 4H), 1.73−1.66 (m, 3H), 1.38−1.31 (m, 3H).
^{13}C NMR (CDCl$_3$, 126 MHz): δ = 160.96, 160.14, 142.31, 142.21, 142.20, 142.16, 128.62, 127.79, 125.54 (q, J = 3 Hz), 120.69, 113.97, 55.33, 35.74, 32.42, 30.95, 26.52, 25.82.
HRMS (ESI) m/z: [M + H]+ calculated for $C_{24}H_{25}F_3NO_2$ 416.1837, found 416.18309.
Rf (hexane/EtOAc 4:1): 0.5.

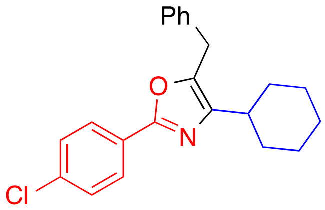

4l

Use the general procedure described above, compound **4l** was obtained from 4-chlorobenzamide (0.2 mmol, 31.10 mg), cyclohexane carboxaldehyde (0.3 mmol, 36.3 uL) and phenylacetylene (0.3 mmol, 34 uL) as a white solid (49.2 mg) in 70% yield.
^{1}H NMR (CDCl$_3$, 500 MHz): δ = 7.93-7.91 (m, 2H), 7.40-7.37 (m, 2H), 7.35-7.30 (m, 2H), 7.28-7.24 (m, 3H), 4.07 (s, 2H), 2.60-2.54 (tt, J = 11.9, 3.6 Hz, 1H), 1.87−1.79 (m, 4H), 1.75−1.66 (m, 3H), 1.41−1.31 (m, 3H).
^{13}C NMR (CDCl$_3$, 126 MHz): δ = 158.85, 144.30, 142.31, 137.80, 135.63, 128.81, 128.65, 128.32, 127.41, 126.65, 126.49, 35.59, 32.40, 31.13, 26.52, 25.85.
HRMS (ESI) m/z: [M + H]+ calculated for $C_{22}H_{23}ClNO$ 352.1468, found 352.14639.

Rf (hexane/EtOAc 4:1): 0.5.

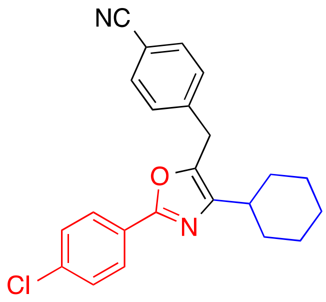

4m

Use the general procedure described above, compound **4m** was obtained from 4-chlorobenzamide (0.2 mmol, 31.10 mg), 4-ethynylbenzonitrile (0.3 mmol, 38.2 mg) and cyclohexane carboxaldehyde (0.3 mmol, 36.3 uL) as a white solid (58.7 mg) in 78% yield.

^{1}H NMR ($CDCl_3$, 500 MHz): δ = 7.91-7.89 (m, 2H), 7.64-7.62 (m, 2H), 7.40-7.38 (m, 2H), 7.35-7.33 (m, 2H), 4.12 (s, 2H), 2.57-2.52 (tt, J = 11.8, 3.6 Hz, 1H), 1.87−1.74 (m, 4H), 1.71−1.67 (m, 3H), 1.38−1.33 (m, 3H).

^{13}C NMR ($CDCl_3$, 126 MHz): δ = 159.29, 143.30, 143.08, 142.56, 135.97, 132.51, 129.08, 128.91, 127.43, 126.19, 118.72, 110.77, 35.64, 32.37, 31.21, 26.44, 25.77.

HRMS (ESI) m/z: [M + H]+ calculated for $C_{23}H_{22}ClN_2O$ 377.1421, found 377.14175.

Rf (hexane/EtOAc 4:1): 0.3.

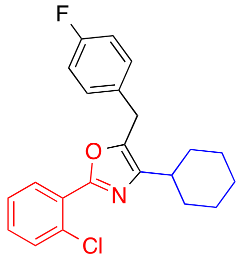

4n

Use the general procedure described above, compound **4n** was obtained from 2-chlorobenzamide (0.2 mmol, 31.10 mg), cyclohexane carboxaldehyde (0.3 mmol, 36.3 uL) and 1-ethynyl-4-fluorobenzene (0.3 mmol, 34.4 uL) as a white solid (53.2 mg) in 72% yield.

^{1}H NMR ($CDCl_3$, 500 MHz): δ = 7.94-7.91 (m, 1H), 7.47-7.44 (m, 1H), 7.33-7.29 (m, 2H), 7.25-7.21 (m, 2H), 7.04-6.99 (m, 2H), 4.05 (s, 2H), 2.61-2.55 (tt, J = 11.7, 3.5 Hz, 1H), 1.85−1.80 (m, 4H), 1.76−1.66 (m, 3H), 1.42−1.30 (m, 3H).

^{13}C NMR ($CDCl_3$, 126 MHz): δ = 162.66, 160.72, 157.86, 144.50 141.78, 133.49, 133.46, 132.33, 130.92, 130.53, 129.88, 129.81, 126.97, 126.65, 115.48, 115.31, 35.55, 32.41, 30.40, 26.50, 25.84.

HRMS (ESI) m/z: [M + H]+ calculated for $C_{22}H_{22}ClFNO$ 370.1374, found 370.13722.

Rf (hexane/EtOAc 4:1): 0.8.

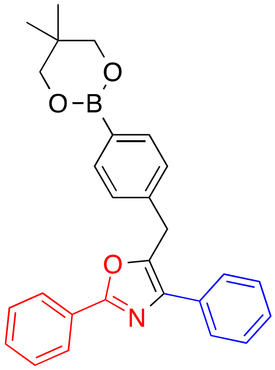

4o

Use the general procedure described above, compound **4o** was obtained from benzamide (0.2 mmol, 24.2 mg), 2-(4-ethynylphenyl)-5,5-dimethyl-1,3,2-dioxaborinane (0.3 mmol, 32 mg) and benzaldehyde (0.3 mmol, 32 uL) as a white solid (42.3 mg) in 50% yield.

^{1}H NMR ($CDCl_3$, 500 MHz): δ = 8.10-8.08 (m, 2H), 7.80-7.78 (m, 2H), 7.76-7.74 (m, 2H), 7.47-7.43 (m, 5H), 7.37-7.36 (m, 1H), 7.32-7.30 (m, 2H), 4.34 (s, 2H), 3.78 (s, 4H), 1.03 (s, 6H).

^{13}C NMR ($CDCl_3$, 126 MHz): δ = 160.17, 145.51, 143.90, 139.73, 137.73, 134.36, 132.07, 130.10, 128.66, 127.59, 127.05, 126.33, 72.31, 32.12, 31.89, 21.90.

HRMS (ESI) m/z: [M + H]+ calculated for $C_{27}H_{27}BNO_3$ 423.2084, found 423.20742.

Rf (hexane/EtOAc 4:1): 0.5.

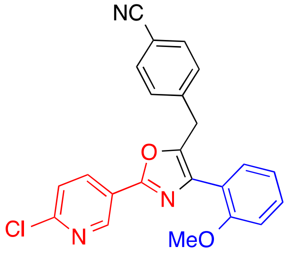

4p

Use the general procedure described above, compound **4p** was obtained from 6-chloronicotinamide (0.2 mmol, 31.31 mg), 4-ethynylbenzonitrile (0.3 mmol, 38.2 mg) and 2-methoxybenzaldehyde (0.3 mmol, 36.3 uL) as a white solid (49.8 mg) in 62% yield.

^{1}H NMR ($CDCl_3$, 500 MHz): δ = 9.04-9.03 (m, 1H), 8.28-8.25 (m, 1H), 7.62-7.59 (m, 3H), 7.43-7.39 (m, 2H), 7.37-7.35 (m, 1H), 7.11-7.08 (m, 1H), 6.99-6.97 (m, 1H), 4.22 (s, 2H), 3.71 (s, 3H).

^{13}C NMR ($CDCl_3$, 126 MHz): δ = 157.39, 156.43, 152.70, 147.44, 146.78, 142.98, 135.99, 135.24, 132.32, 131.11, 130.18, 129.27, 124.38, 122.65, 121.10, 120.06, 118.74, 111.18, 110.67, 55.32, 32.37.

HRMS (ESI) m/z: [M + H]+ calculated for $C_{23}H_{17}ClN_3O_2$ 402.1009, found 401.10071.

Rf (hexane/EtOAc 4:1): 0.5.

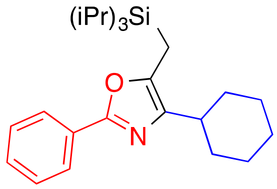

4q

Use the general procedure described above, compound **4q** was obtained from benzamide (0.2 mmol, 24.2 mg), cyclohexane carboxaldehyde (0.3 mmol, 36.3 uL) and triisopropylsilyl acetylene (0.3 mmol, 67.3 uL) as a white solid (33.4 mg) in 42% yield.
^{1}H NMR (CDCl$_3$, 500 MHz): 7.97-7.95 (m, 2H), 7.44-7.41 (m, 2H), 7.39-7.38 (m, 1H), 2.51-2.46 (tt, J = 11.7, 3.5 Hz, 1H), 2.13 (s, 2H), 1.87−1.85 (m, 2H), 1.78−1.69 (m, 5H), 1.37−1.35 (m, 3H), 1.19-1.16 (m, 3H), 1.10-1.08 (d, J = 5.4 Hz, 18H).
^{13}C NMR (CDCl$_3$, 126 MHz): δ = 158.29, 144.80, 129.19, 128.62, 128.59, 125.75, 125.70, 35.58, 32.26, 26.65, 25.90, 18.56, 11.30, 6.70.
HRMS (ESI) m/z: [M + H]+ calculated for $C_{25}H_{40}NOSi$ 398.2879, found 398.28751.
Rf (hexane/EtOAc 4:1): 0.8.

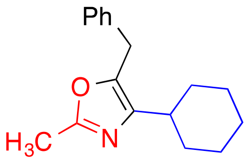

4r

Use the general procedure described above, compound **4r** was obtained from acetamide (0.2 mmol, 17.4 mg), cyclohexane carboxaldehyde (0.3 mmol, 36.3 uL) and phenylacetylene (0.3 mmol, 34 uL) as a white solid (26.5 mg) in 52% yield.
to yield the corresponding oxazole **4r** as a yellowish solid (26.5 mg, 52%).
^{1}H NMR (CDCl$_3$, 500 MHz): 7.33-7.30 (m, 2H), 7.26-7.23 (m, 1H), 7.20-7.19 (m, 2H), 3.94 (s, 2H), 2.50-2.44 (tt, J = 11.9, 3.5 Hz, 1H), 2.37 (s, 3H), 1.84−1.82 (m, 2H), 1.75−1.71 (m, 3H), 1.65−1.58 (m, 2H), 1.37-1.27 (m, 3H).
^{13}C NMR (CDCl$_3$, 126 MHz): δ = 159.56, 143.34, 140.23, 138.08, 128.56, 128.29, 126.53, 35.24, 30.89, 26.55, 25.84, 14.01.
HRMS (ESI) m/z: [M + H]+ calculated for $C_{17}H_{22}NO$ 255.1623, found 255.16241.
Rf (hexane/EtOAc 4:1): 0.7.

4 PDF file of ¹H NMR and ¹³C NMR spectra for new compounds

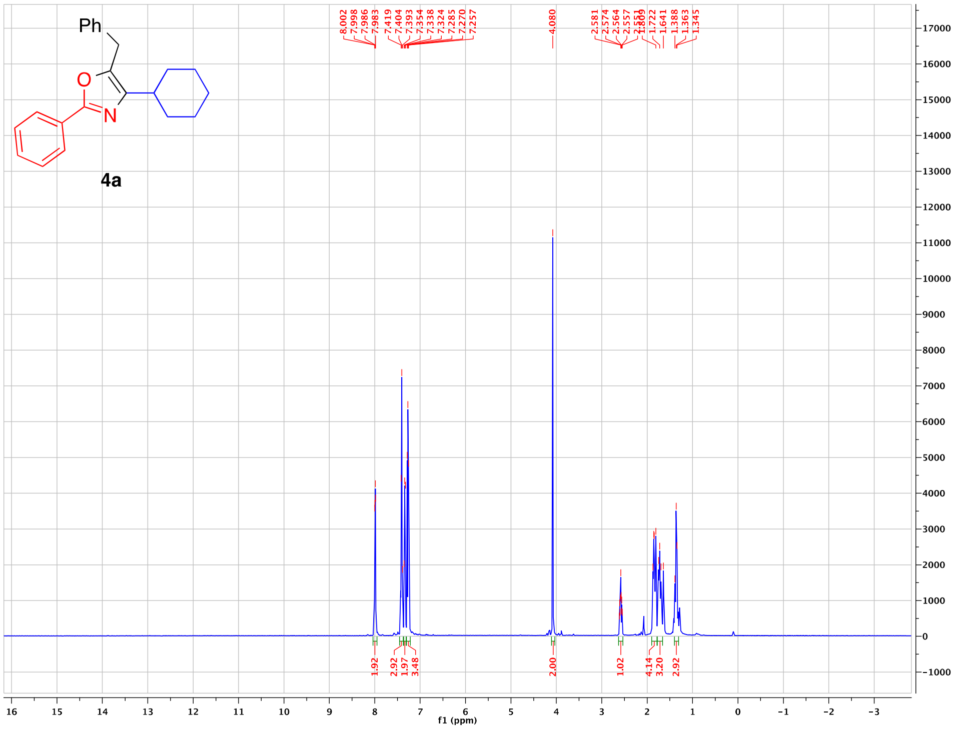

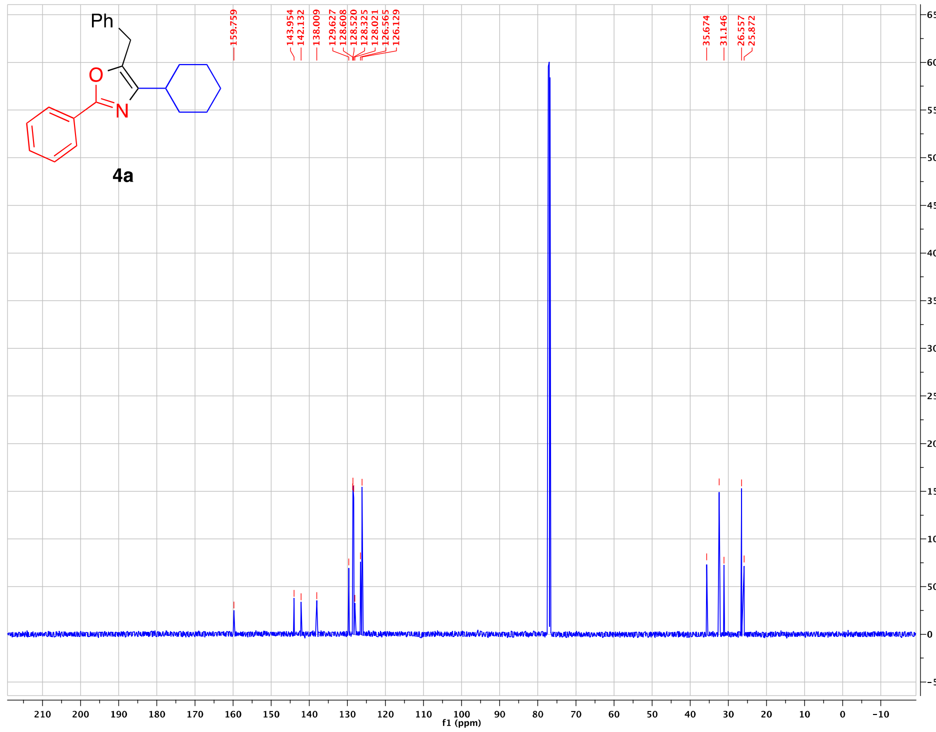
Ph
O
N
4a
159.759
143.954
142.132
138.009
129.627
128.608
128.520
128.325
128.021
126.565
126.129
35.674
31.146
26.557
25.872
f1 (ppm)

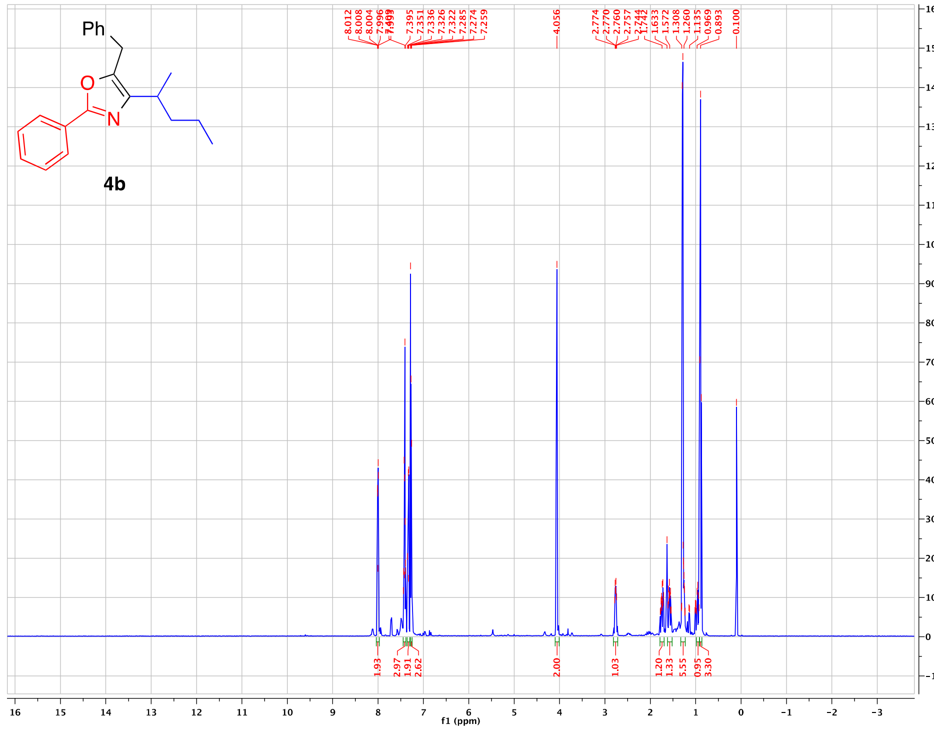
Ph
O
N
4b
4.056
0.100
1.93
2.97
1.91
2.62
2.00
1.03
1.20
1.33
5.55
0.95
3.30
f1 (ppm)

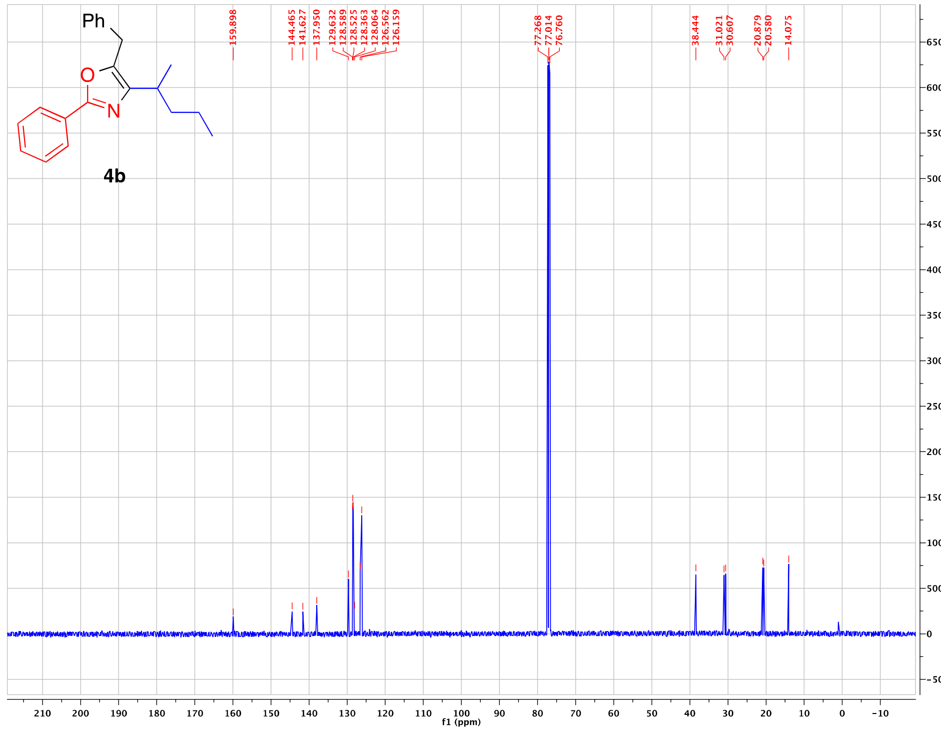
Ph
O
N
4b
159.898
144.465
141.627
137.950
129.632
128.589
128.525
128.363
128.064
126.562
126.159
77.268
77.014
76.760
38.444
31.021
30.607
20.879
20.580
14.075
f1 (ppm)

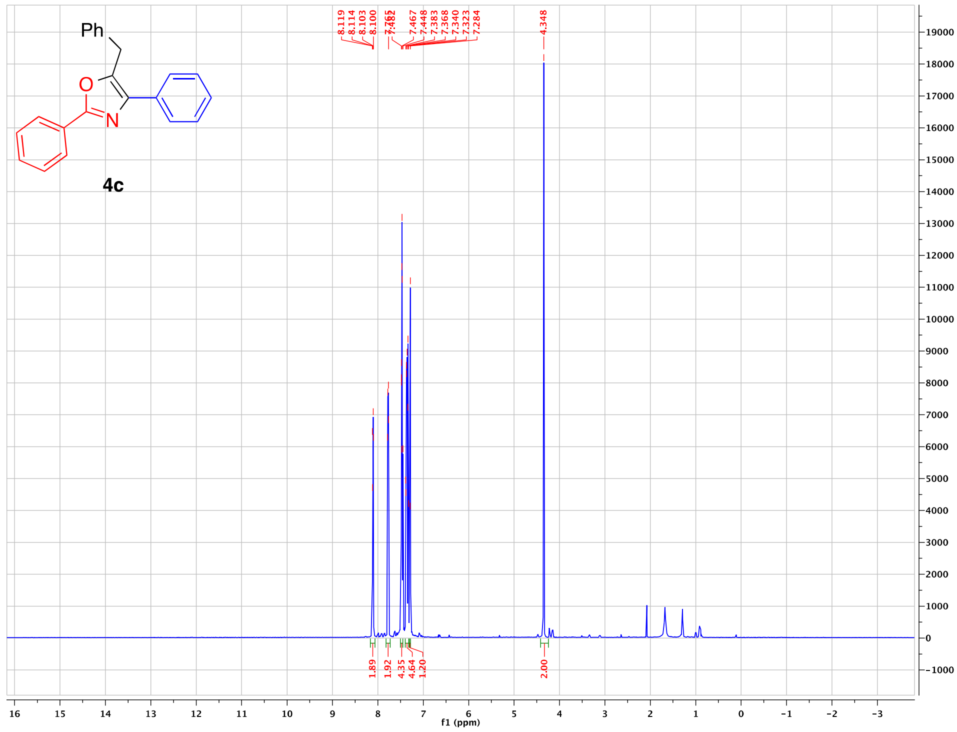
Ph
O
N
4c
8.119
8.114
8.103
8.100
7.482
7.467
7.448
7.383
7.368
7.340
7.323
7.284
4.348
1.89
1.92
4.35
4.64
1.20
2.00
f1 (ppm)

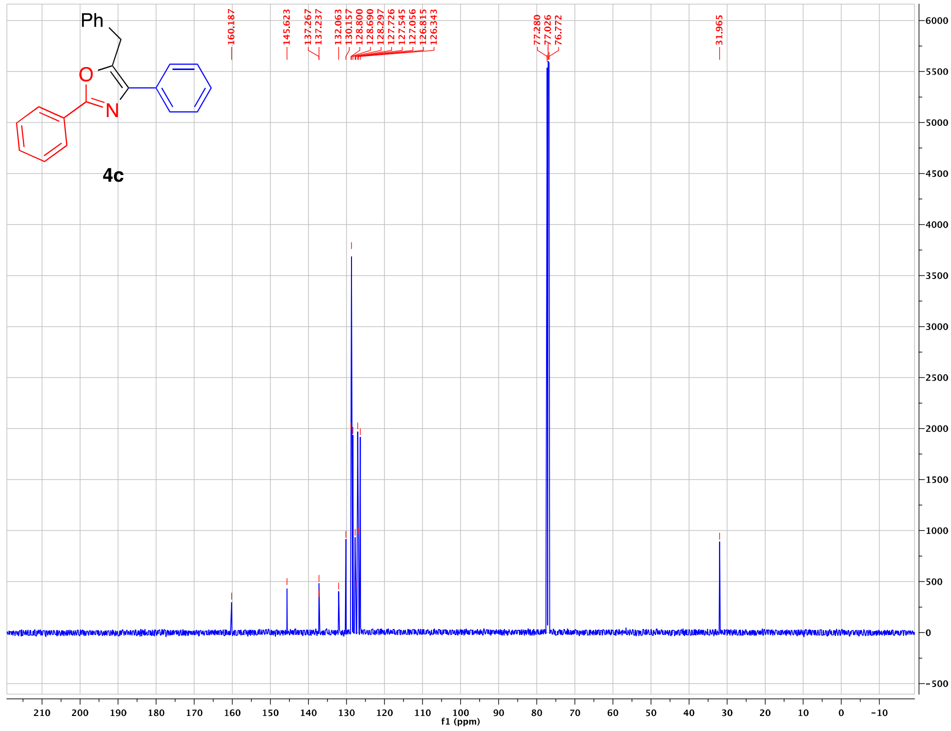
4c
Ph
O
N
160.187
145.623
137.267
137.237
132.063
130.157
128.800
128.690
128.297
127.726
127.545
127.056
126.815
126.343
77.280
77.026
76.772
31.965
f1 (ppm)

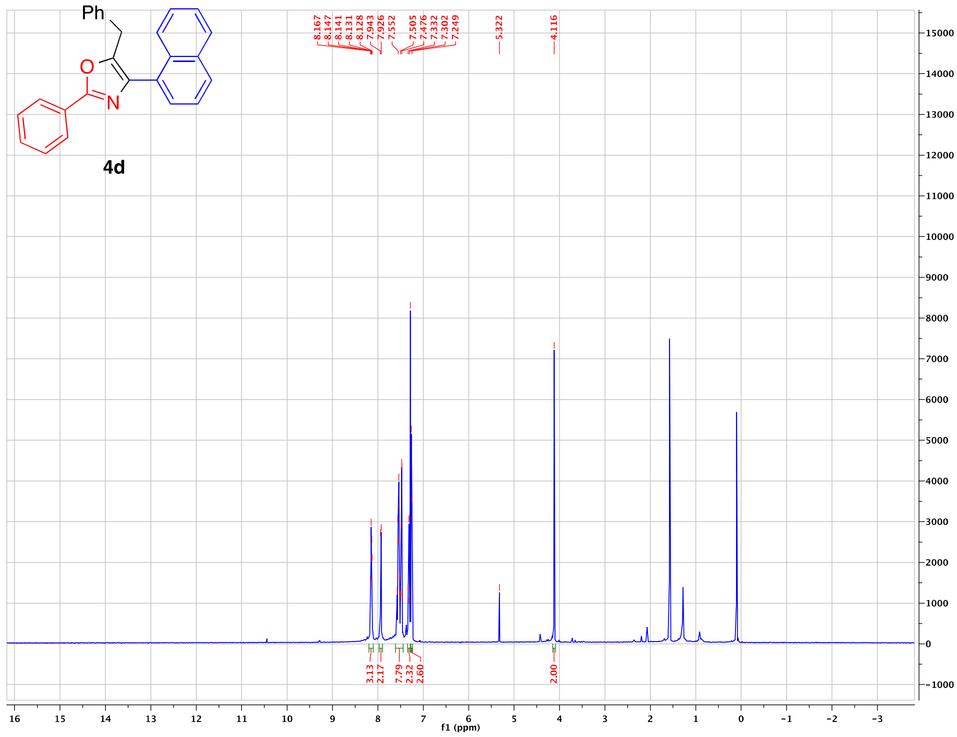
4d
Ph
O
N
8.167
8.147
8.141
8.131
8.128
7.943
7.926
7.552
7.505
7.476
7.332
7.302
7.249
5.322
4.116
3.13
2.17
7.79
2.32
2.60
2.00
f1 (ppm)

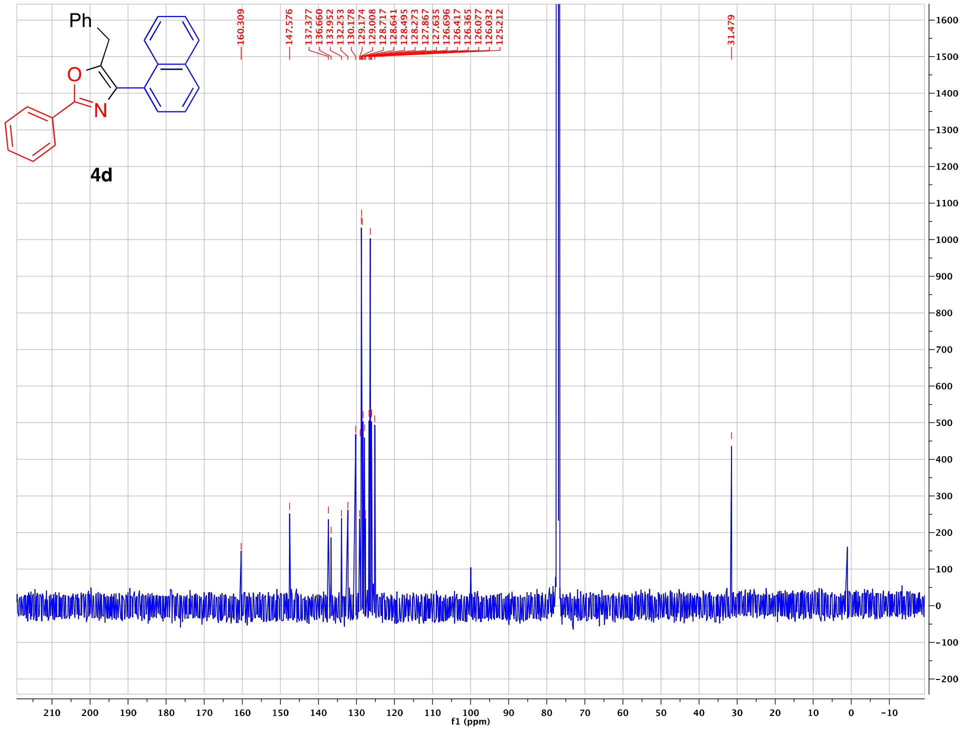

Ph
O
N
4d
160.309
147.576
137.377
136.660
133.952
132.253
130.178
129.174
129.008
128.717
128.641
128.495
128.273
127.867
127.635
126.696
126.417
126.365
126.077
126.032
125.212
31.479
f1 (ppm)

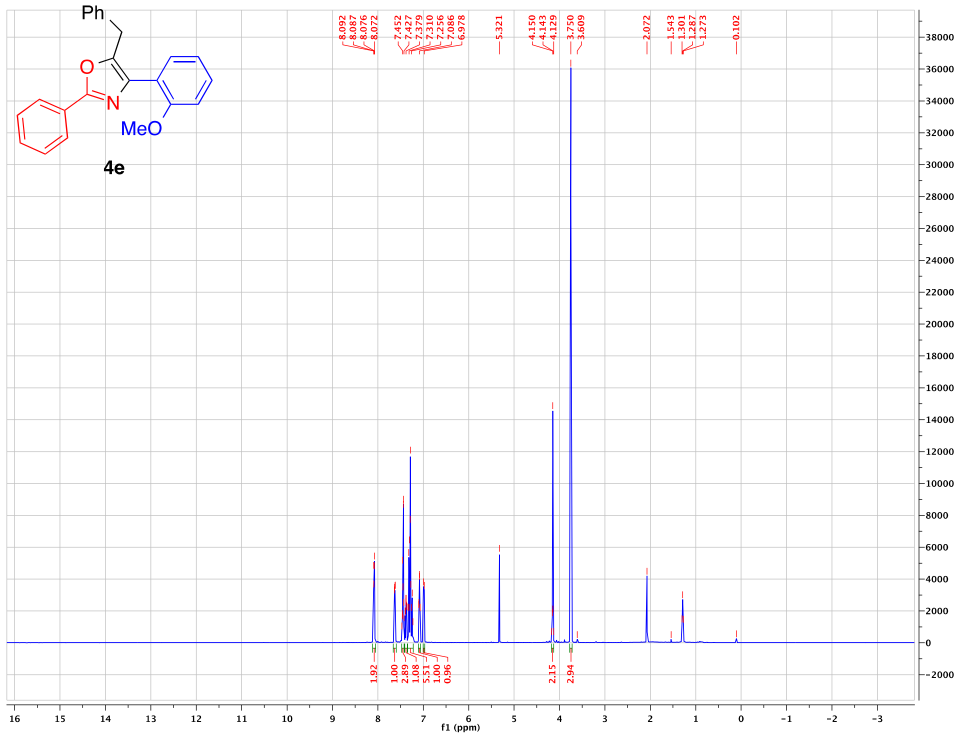

Ph
O
N
MeO
4e
8.092
8.087
8.076
8.072
7.452
7.427
7.379
7.310
7.256
7.086
6.978
5.321
4.150
4.143
4.129
3.750
3.609
2.072
1.543
1.301
1.287
1.273
0.102
1.92
1.00
2.89
1.08
5.51
1.00
0.96
2.15
2.94
f1 (ppm)

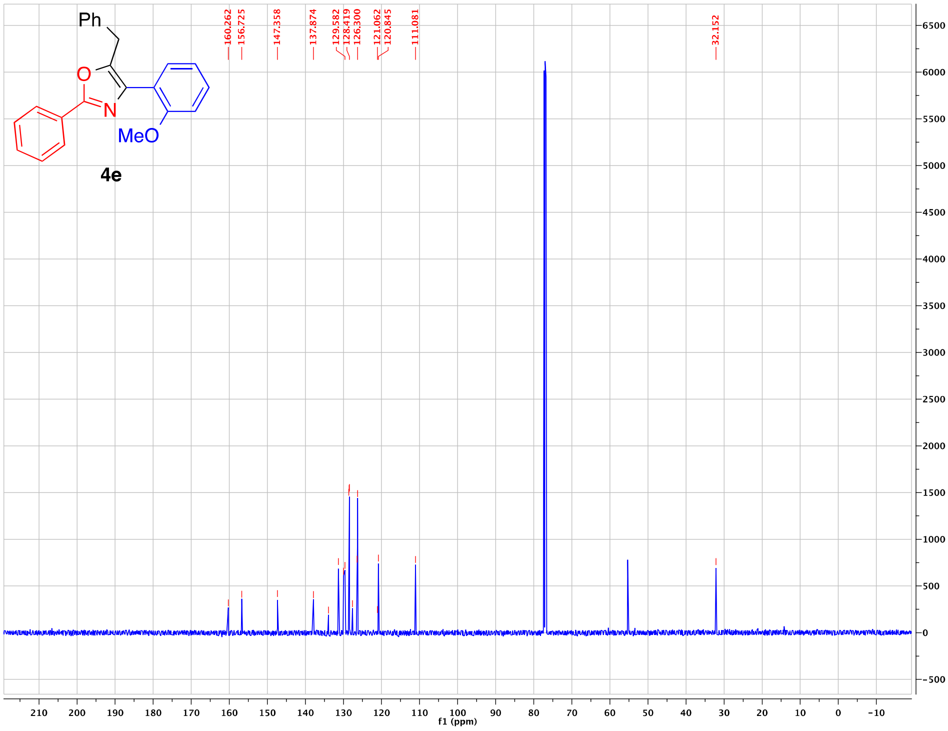
Ph
O
N
MeO
160.262
156.725
147.358
137.874
129.582
128.419
126.300
121.062
120.845
111.081
32.152
f1 (ppm)

4e

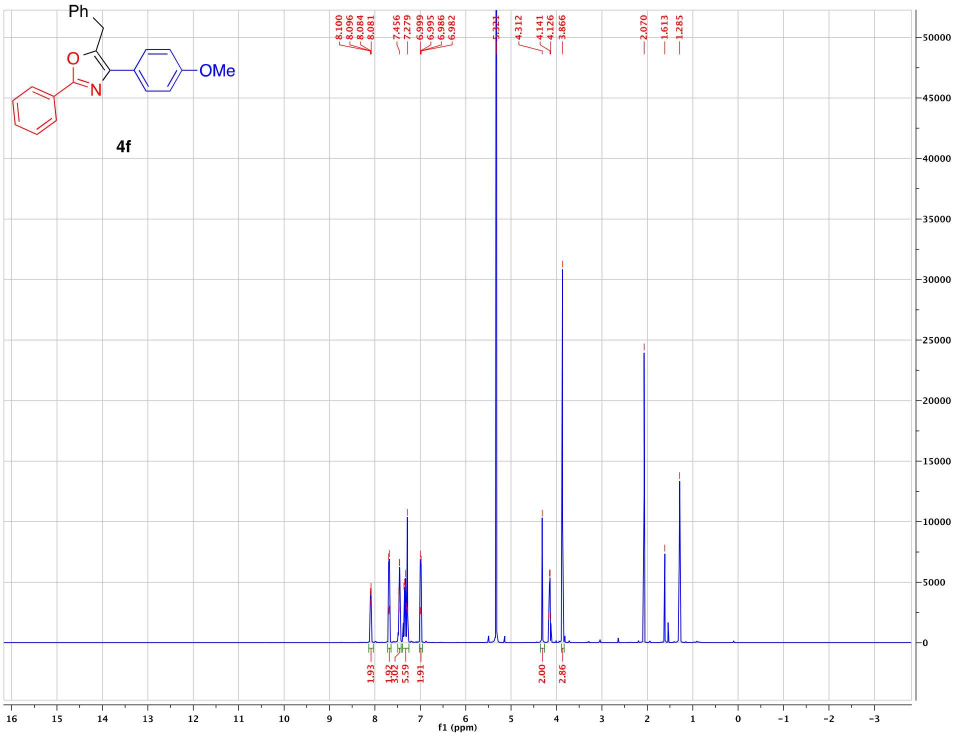
Ph
O
N
OMe
8.100
8.096
8.084
8.081
7.456
7.279
6.999
6.995
6.986
6.982
4.312
4.141
4.126
3.866
2.070
1.613
1.285
1.93
1.92
3.02
5.59
1.91
2.00
2.86
f1 (ppm)

4f

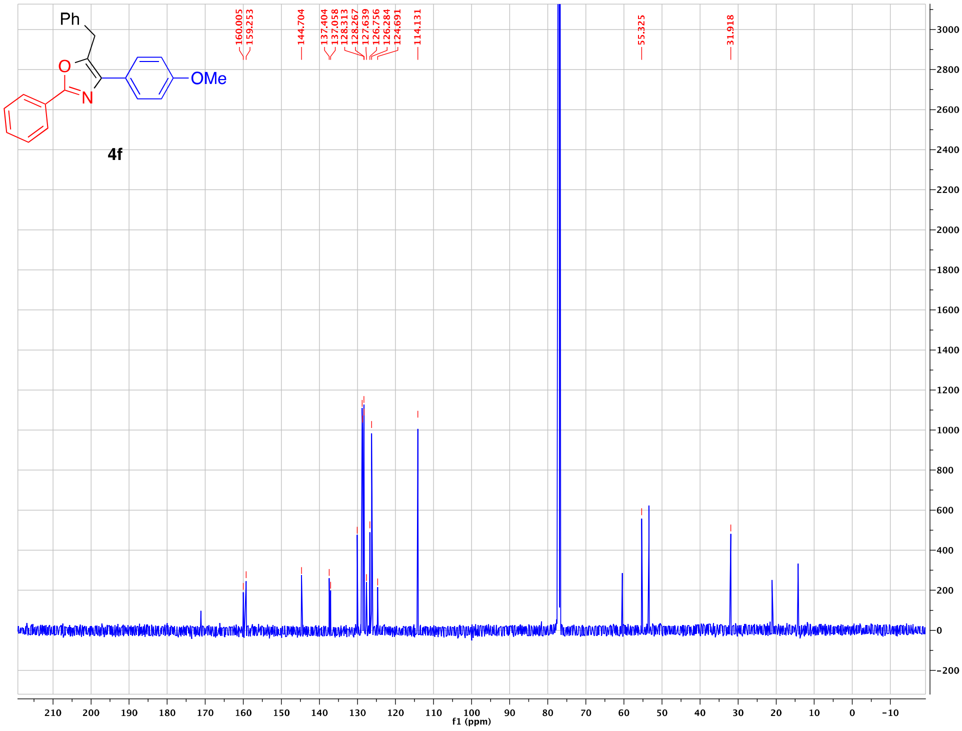

Ph
O
N
OMe
4f
160.005
159.253
144.704
137.404
137.058
128.313
128.267
127.639
126.756
126.284
124.691
114.131
55.325
31.918
f1 (ppm)

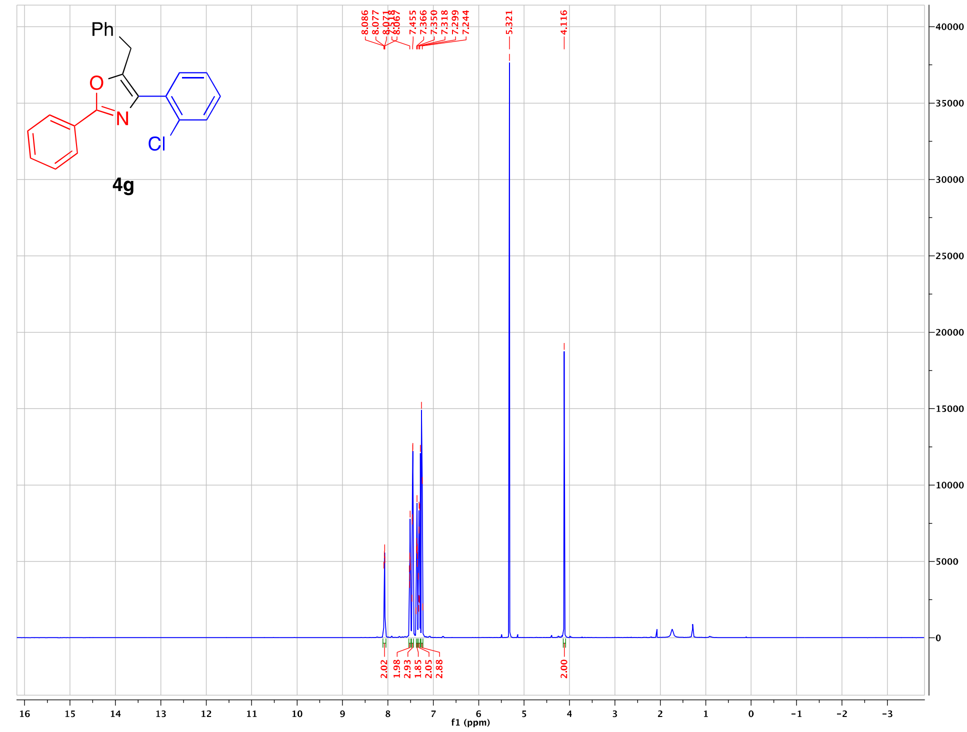

Ph
O
N
Cl
4g
8.086
8.077
8.071
8.067
7.455
7.366
7.350
7.318
7.299
7.244
5.321
4.116
2.02
1.98
2.93
1.85
2.05
2.88
2.00
f1 (ppm)

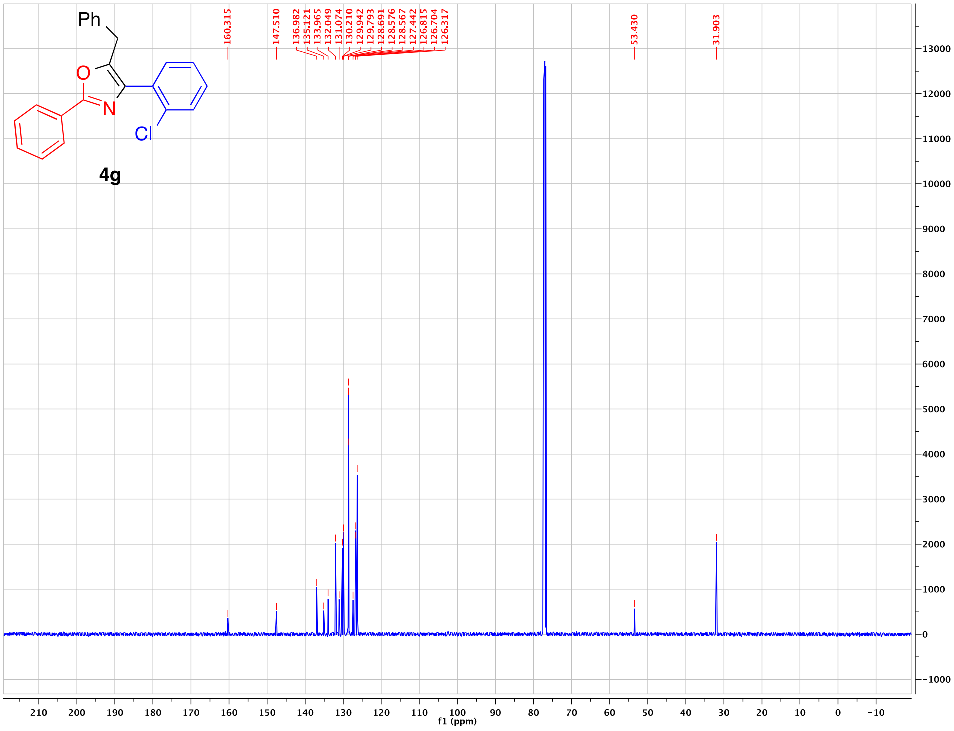

Ph
O
N
Cl
4g
160.315
147.510
136.982
135.121
133.965
132.049
131.074
130.210
129.942
129.793
128.691
128.576
128.567
127.442
126.815
126.704
126.317
53.430
31.903
f1 (ppm)

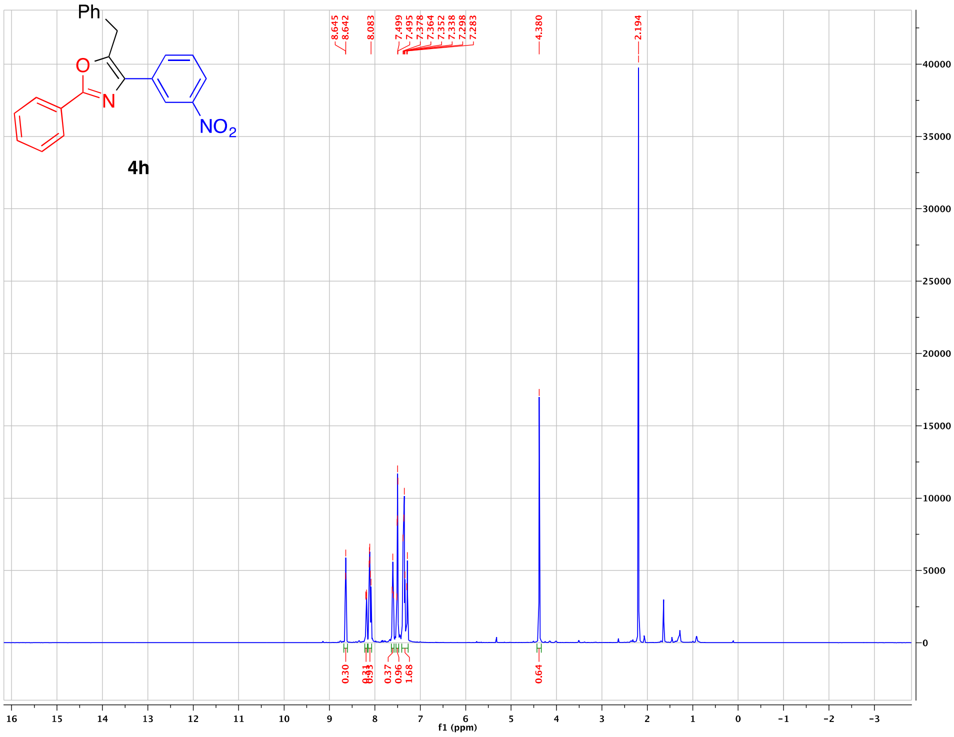

Ph
O
N
NO2
4h
8.645
8.642
8.083
7.499
7.495
7.378
7.364
7.352
7.338
7.298
7.283
4.380
2.194
0.30
0.37
0.96
1.68
0.64
f1 (ppm)

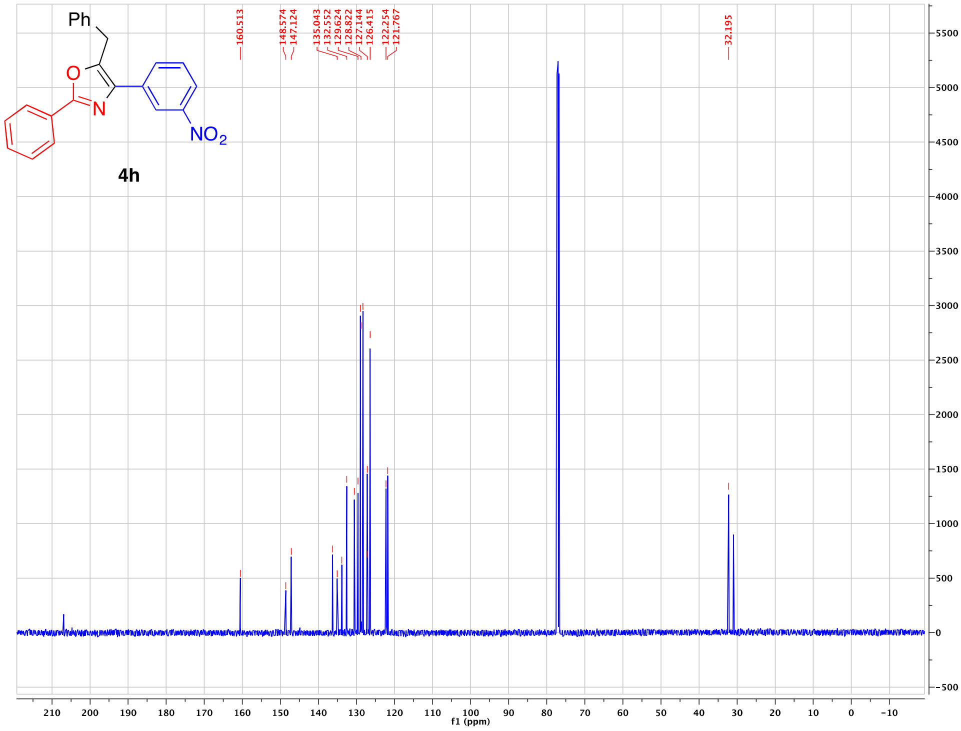
Ph
O
N
NO2
4h
160.513
148.574
147.124
135.043
132.552
129.624
128.822
127.144
126.415
122.254
121.767
32.195
f1 (ppm)

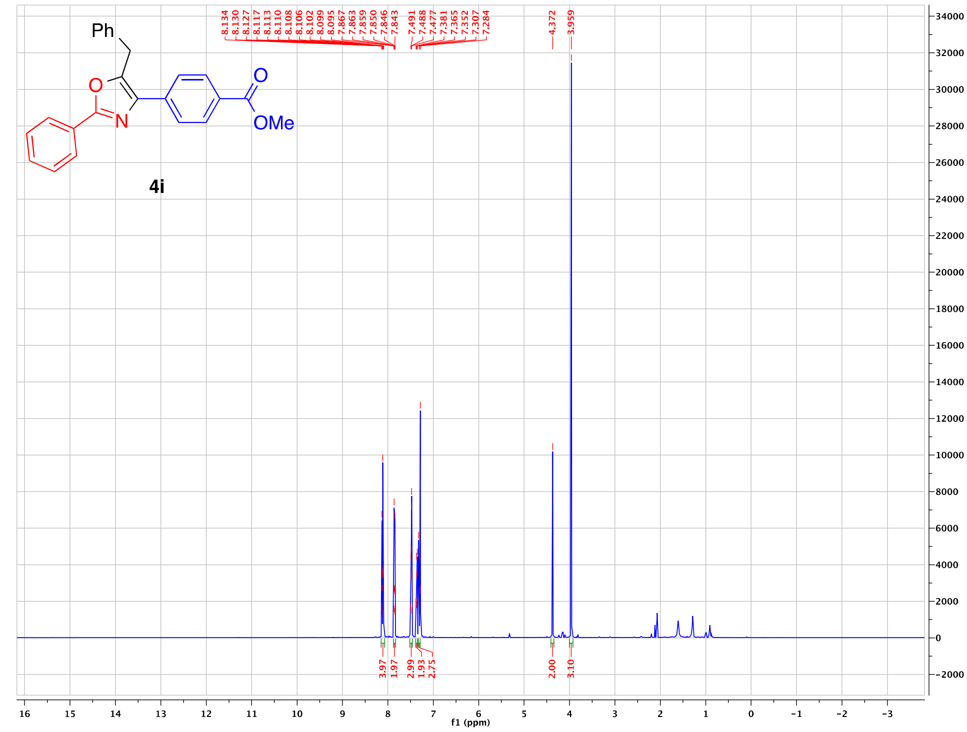
Ph
O
N
O
OMe
4i
8.134
8.130
8.127
8.117
8.113
8.110
8.108
8.106
8.102
8.099
8.095
7.867
7.863
7.859
7.850
7.846
7.843
7.491
7.488
7.477
7.381
7.365
7.352
7.307
7.284
4.372
3.959
3.97
1.97
2.99
1.93
2.75
2.00
3.10
f1 (ppm)

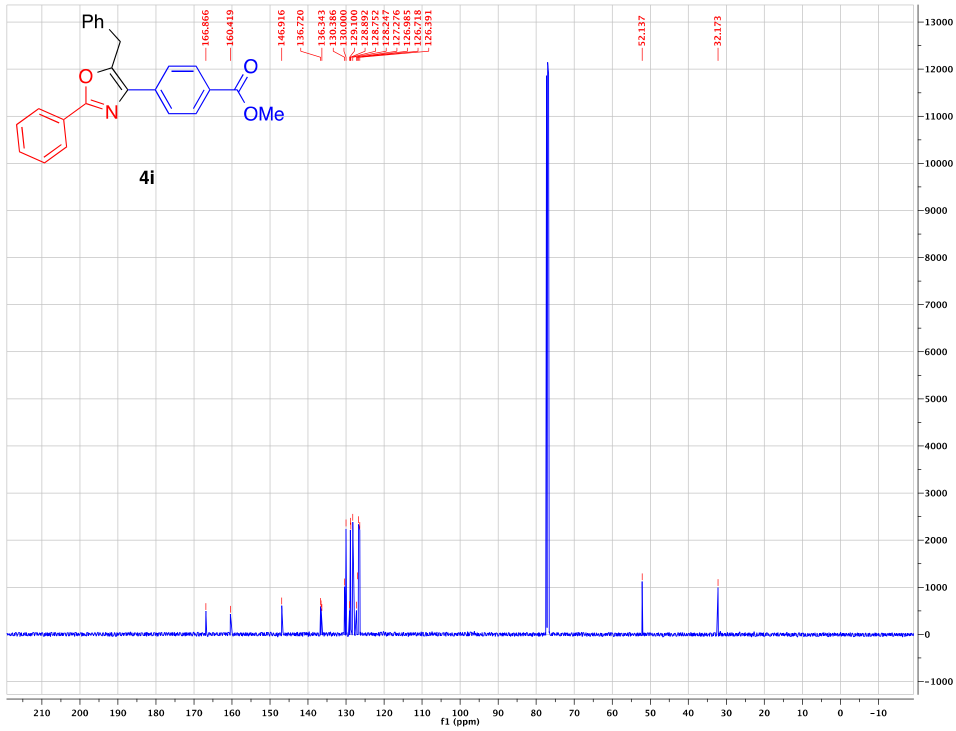

4i

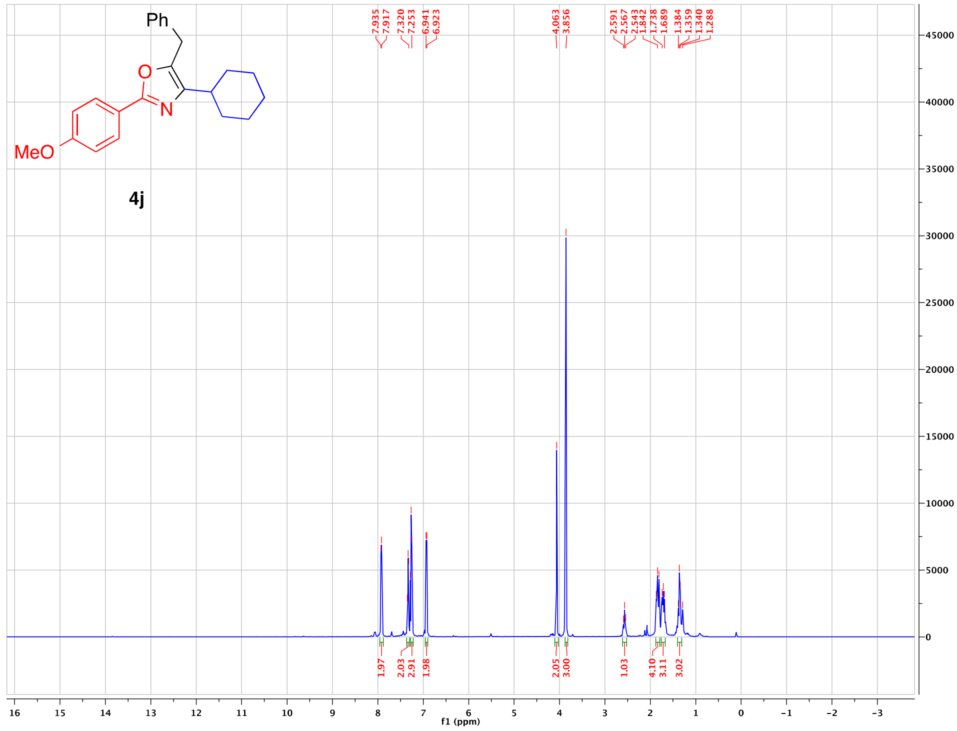

4j

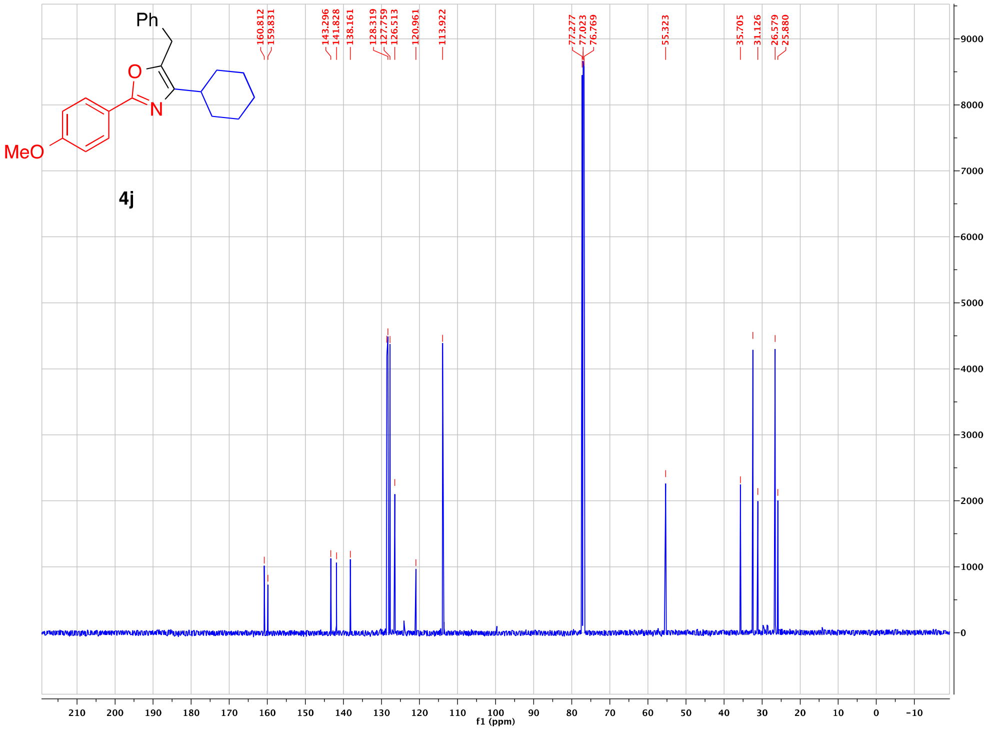
Ph
O
N
MeO
4j
160.812
159.831
143.296
141.828
138.161
128.319
127.759
126.513
120.961
113.922
77.277
77.023
76.769
55.323
35.705
31.126
26.579
25.880
f1 (ppm)

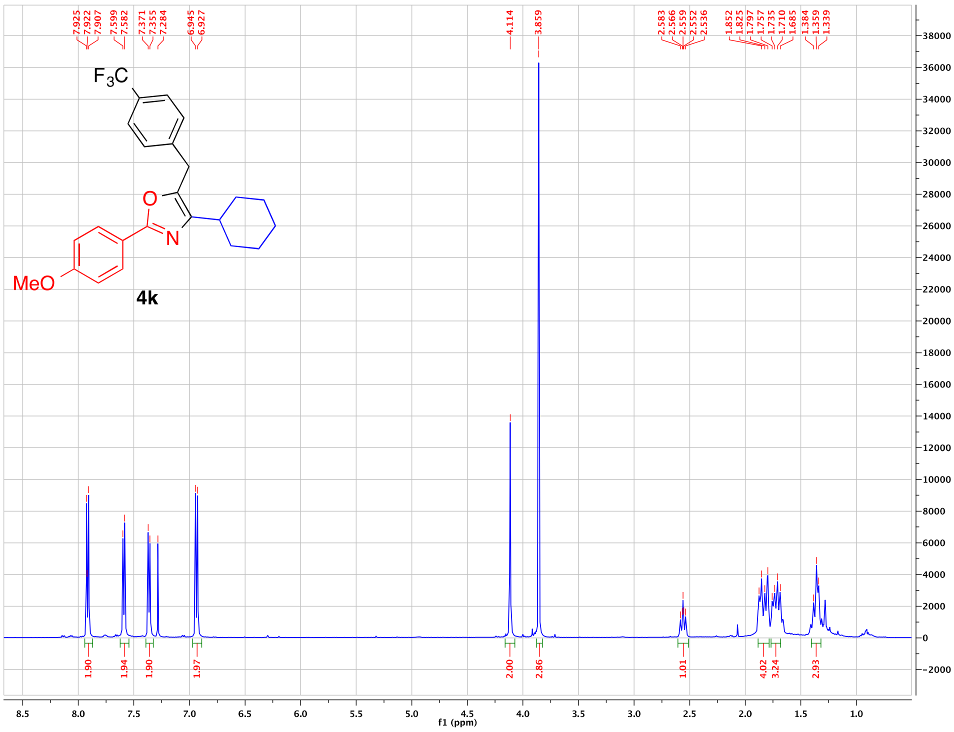
F_3C
O
N
MeO
4k
7.925
7.922
7.907
7.599
7.582
7.371
7.355
7.284
6.945
6.927
4.114
3.859
2.583
2.566
2.559
2.552
2.536
1.852
1.825
1.797
1.757
1.735
1.710
1.685
1.384
1.359
1.339
1.90
1.94
1.90
1.97
2.00
2.86
1.01
4.02
3.24
2.93
f1 (ppm)

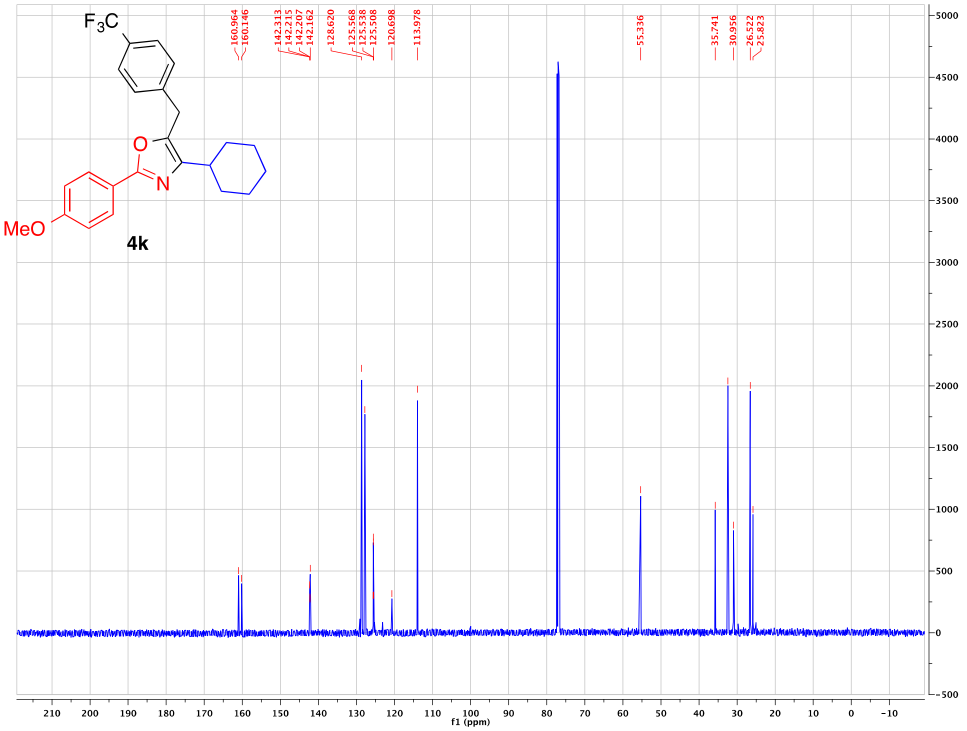
F3C
O
N
MeO
4k
160.964
160.146
142.313
142.215
142.207
142.162
128.620
125.568
125.538
125.508
120.698
113.978
55.336
35.741
30.956
26.522
25.823
f1 (ppm)

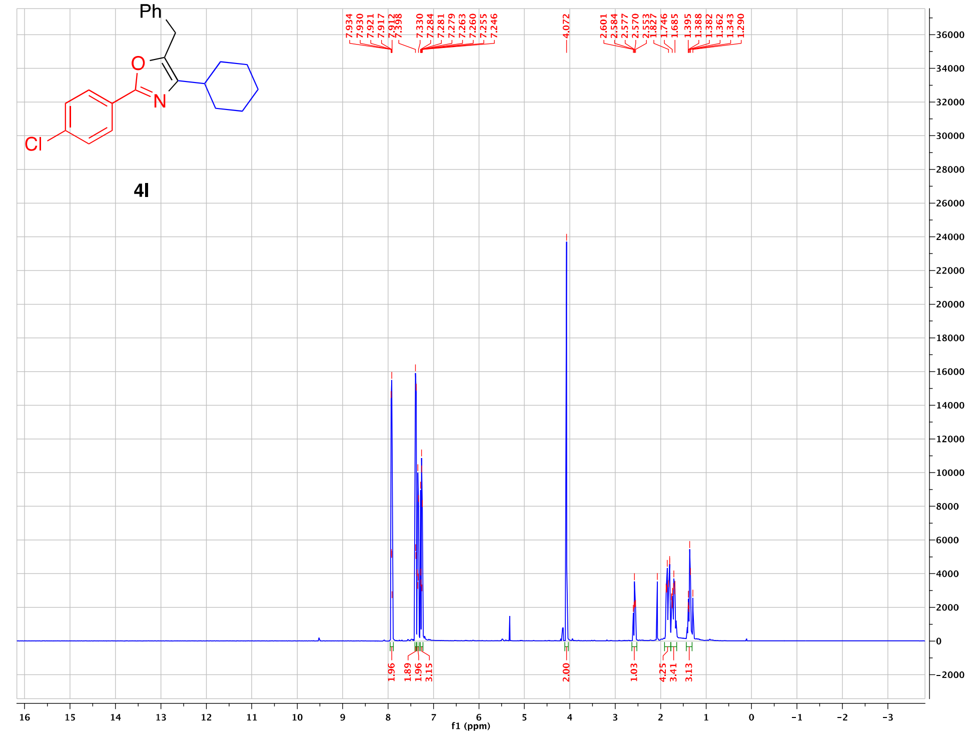
Ph
O
N
Cl
4l
7.934
7.930
7.921
7.917
7.912
7.398
7.330
7.284
7.281
7.279
7.263
7.260
7.255
7.246
4.072
2.601
2.584
2.577
2.570
2.553
1.827
1.746
1.685
1.395
1.388
1.382
1.362
1.343
1.290
1.96
1.89
1.96
3.15
2.00
1.03
4.25
3.41
3.13
f1 (ppm)

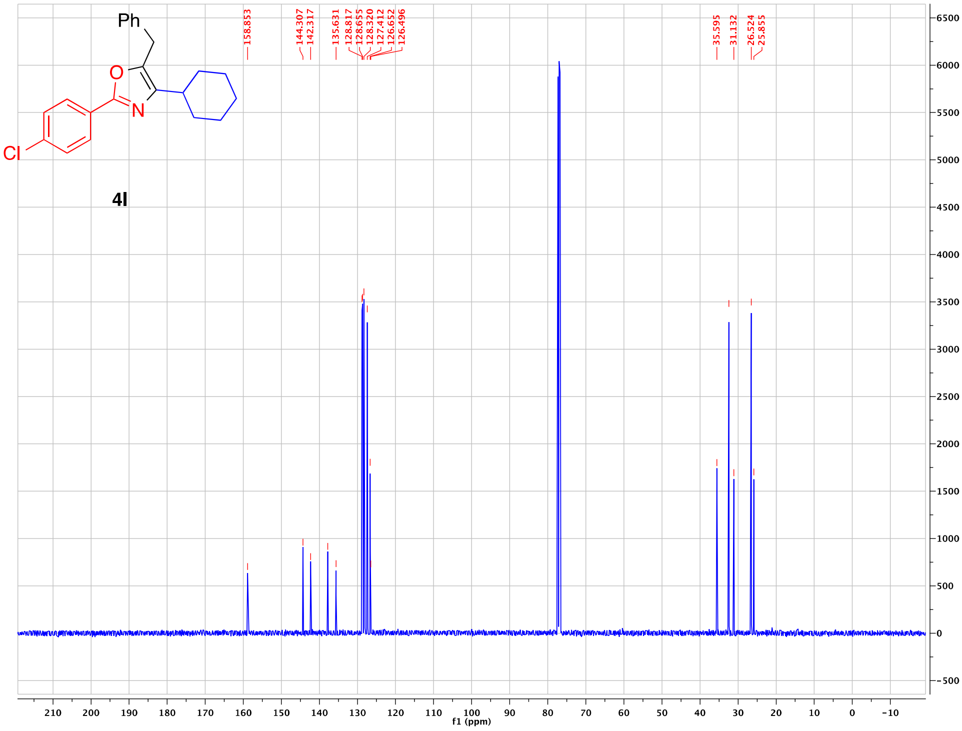

Ph
O
N
Cl
4l
f1 (ppm)

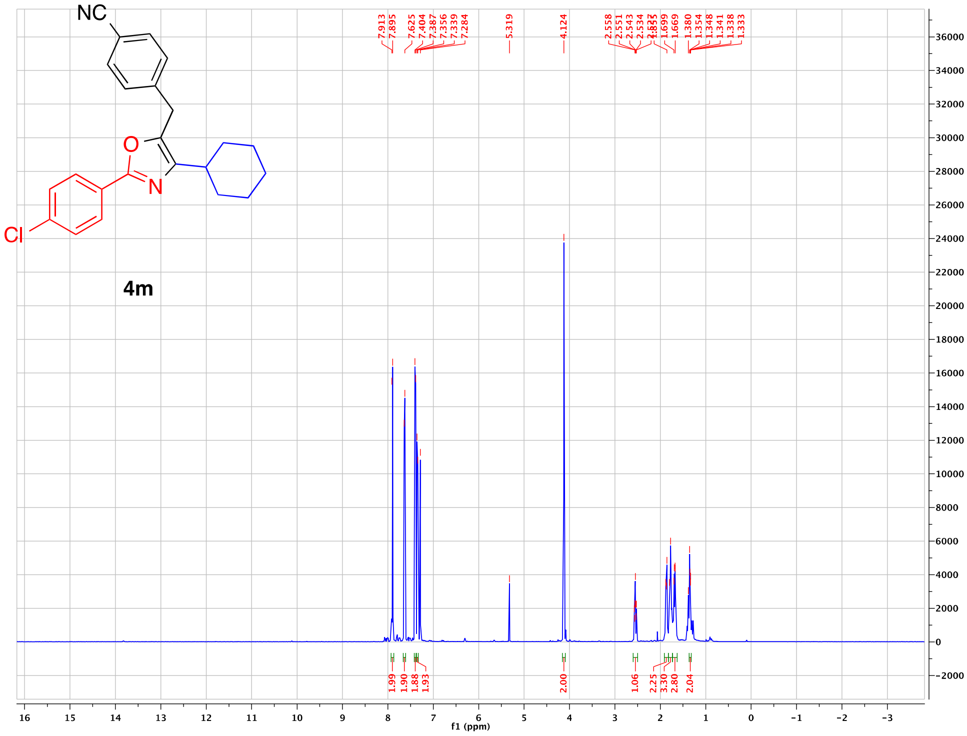

NC
O
N
Cl
4m
f1 (ppm)

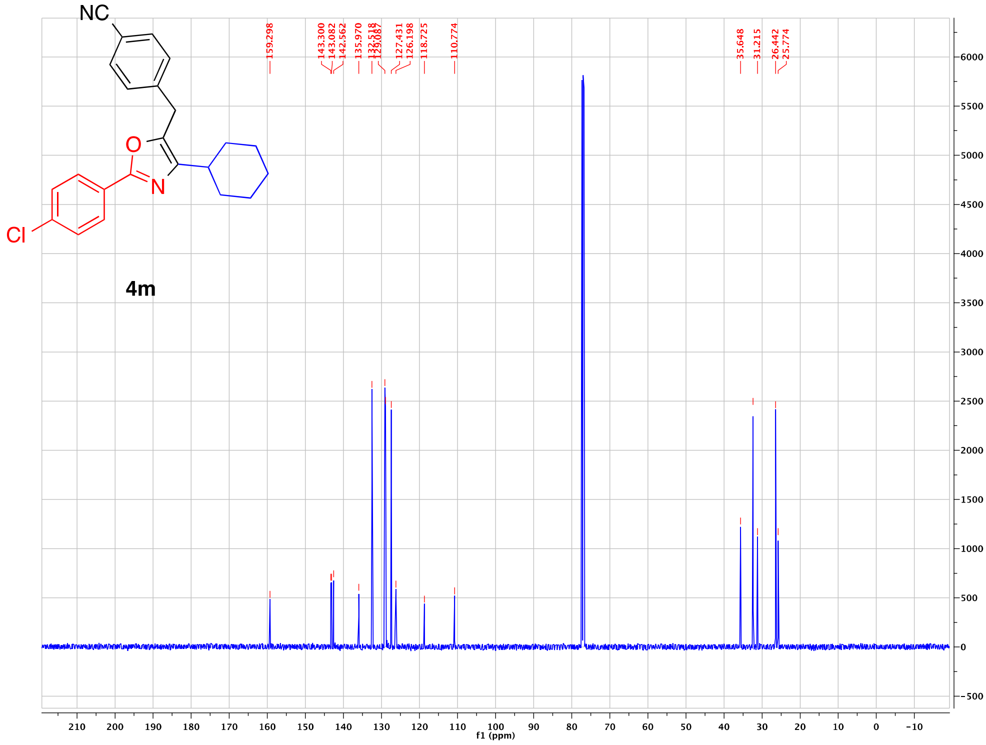

4m

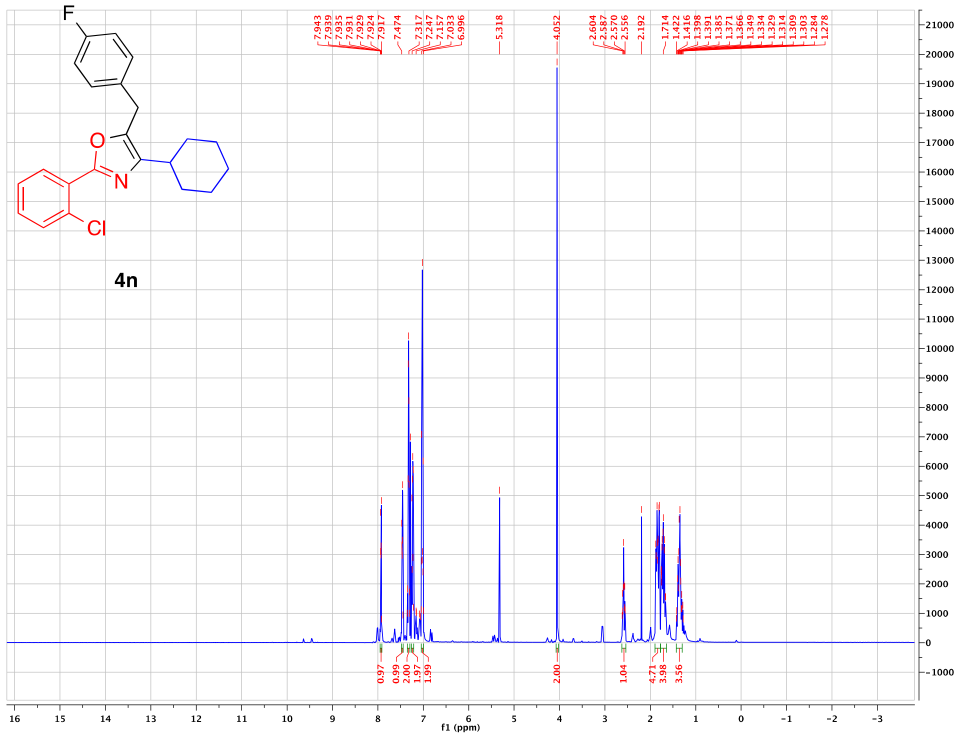

4n

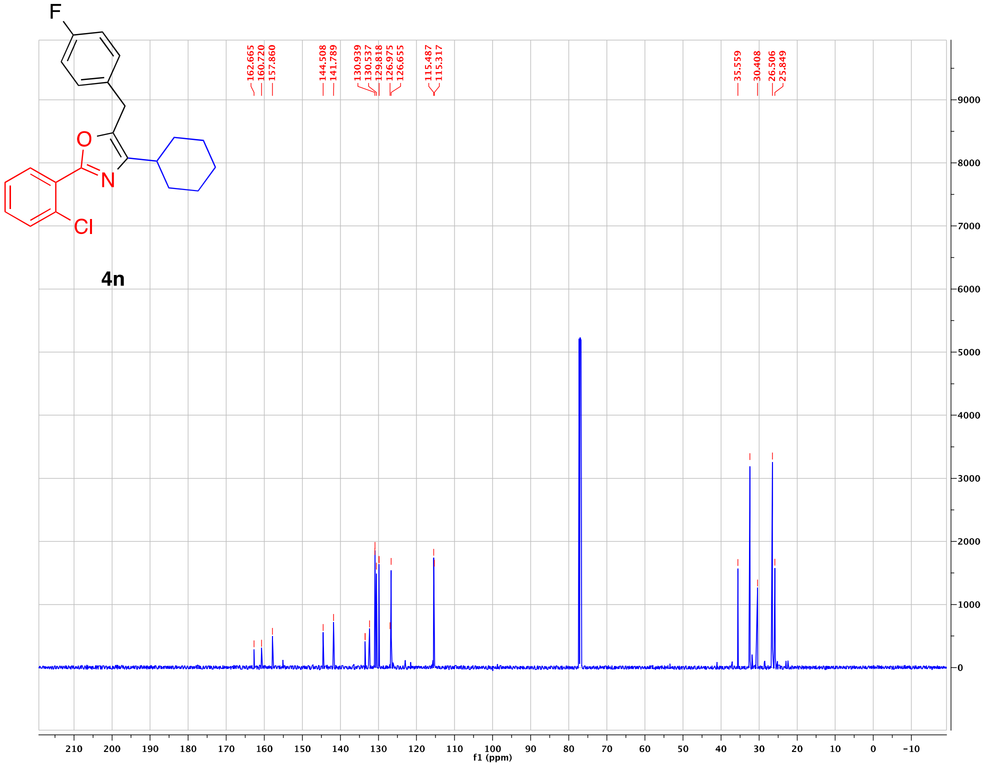

4n

162.665
160.720
157.860
144.508
141.789
130.939
130.537
129.818
126.975
126.655
115.487
115.317
35.559
30.408
26.506
25.849
f1 (ppm)

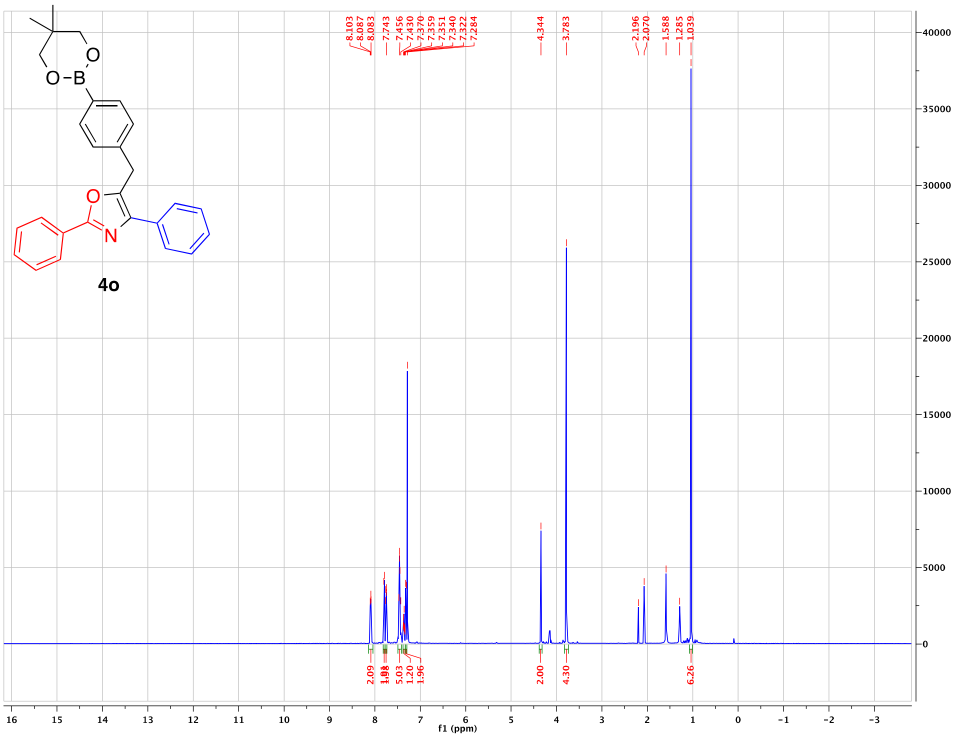

4o

8.103
8.087
8.083
7.743
7.456
7.430
7.370
7.359
7.351
7.340
7.322
7.284
4.344
3.783
2.196
2.070
1.588
1.285
1.039
2.09
5.03
1.20
1.96
2.00
4.30
6.26
f1 (ppm)

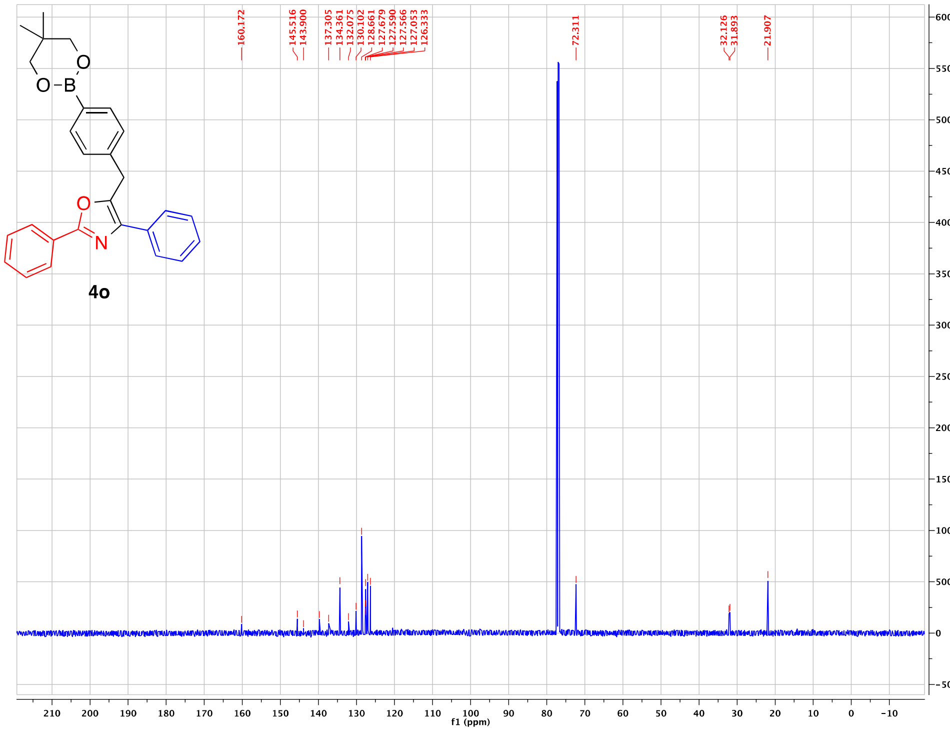
4o
160.172
145.516
143.900
137.305
134.361
132.075
130.102
128.661
127.679
127.590
127.566
127.053
126.333
72.311
32.126
31.893
21.907
f1 (ppm)

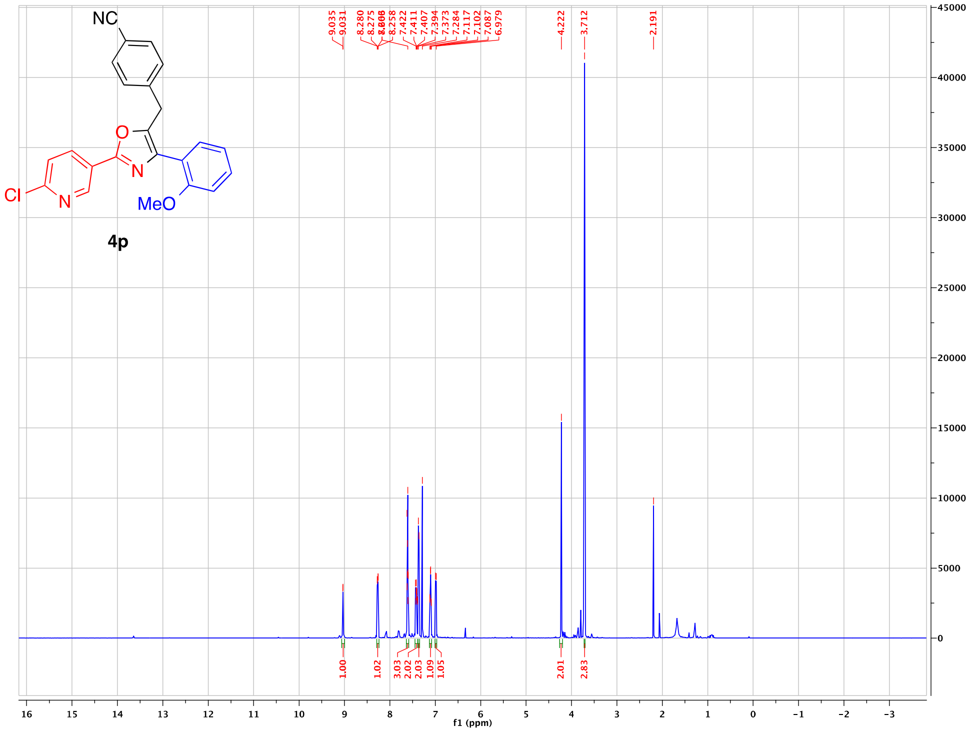
NC
O
N
Cl
N
MeO
4p
9.035
9.031
8.280
8.275
8.258
7.422
7.411
7.407
7.394
7.373
7.284
7.117
7.102
7.087
6.979
4.222
3.712
2.191
1.00
1.02
3.03
2.02
2.03
1.09
1.05
2.01
2.83
f1 (ppm)

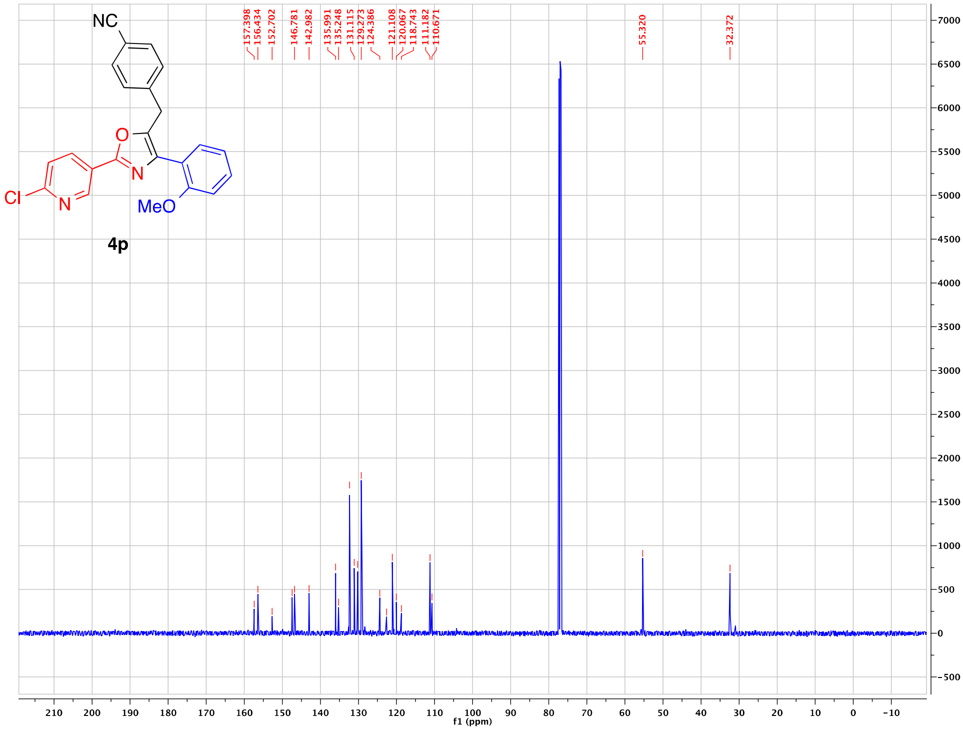

NC
O
N
Cl
N
MeO
4p
157.398
156.434
152.702
146.781
142.982
135.991
135.248
131.115
129.273
124.386
121.108
120.067
118.743
111.182
110.671
55.320
32.372
f1 (ppm)

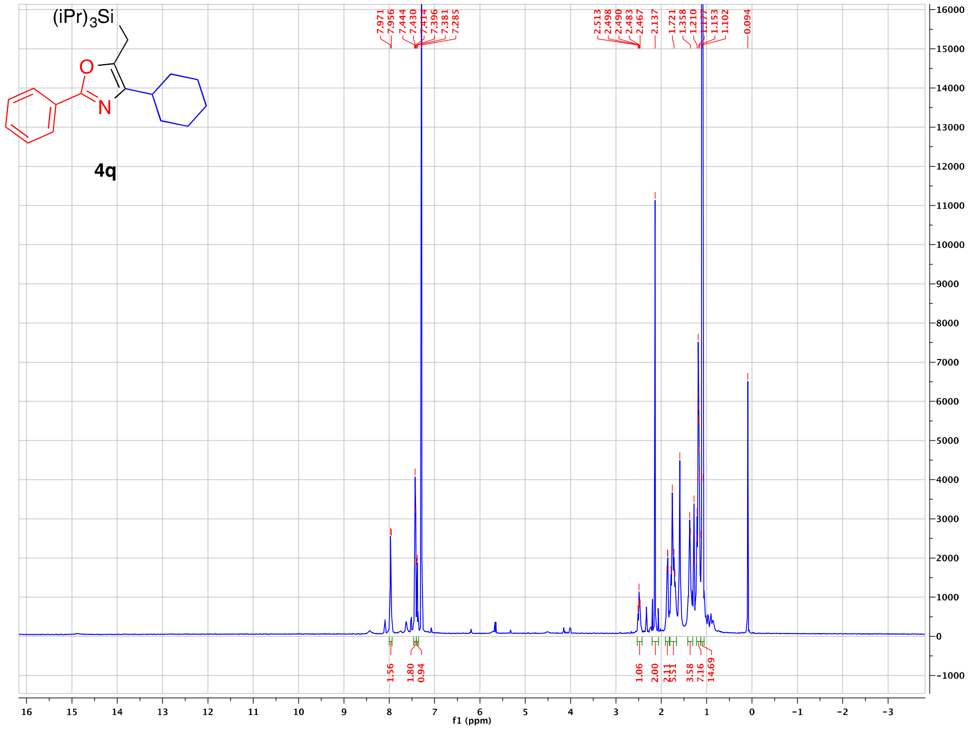

(iPr)3Si
O
N
4q
7.971
7.956
7.444
7.430
7.414
7.396
7.381
7.285
2.513
2.498
2.490
2.483
2.467
2.137
1.721
1.358
1.210
1.177
1.153
1.102
0.094
1.56
1.80
0.94
1.06
2.00
2.11
5.51
3.58
7.16
14.69
f1 (ppm)

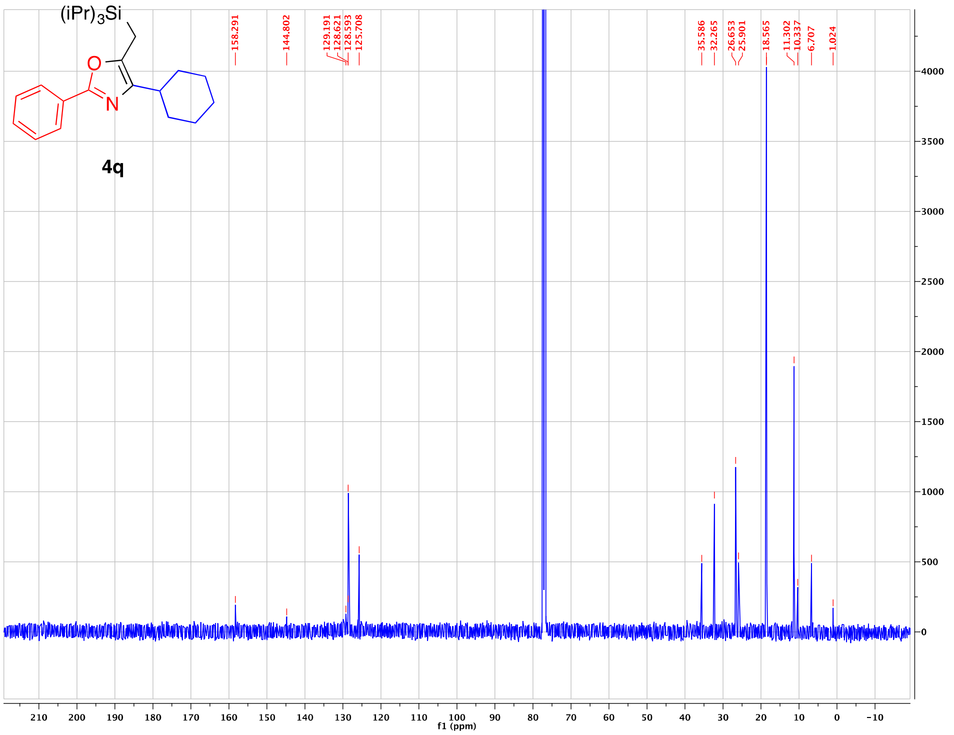
(iPr)3Si
O
N
4q
158.291
144.802
129.191
128.621
128.593
125.708
35.586
32.265
26.653
25.901
18.565
11.302
10.337
6.707
1.024
f1 (ppm)

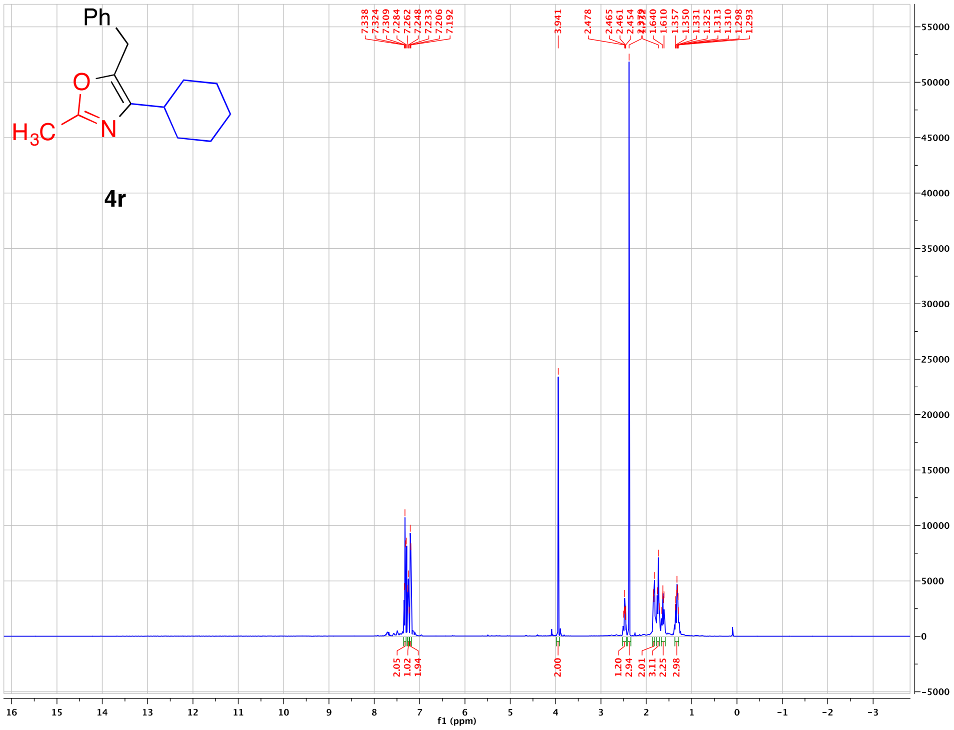
Ph
O
N
H3C
4r
7.338
7.324
7.309
7.284
7.262
7.248
7.233
7.206
7.192
3.941
2.478
2.465
2.461
2.454
2.00
1.20
2.94
2.01
3.11
2.25
2.98
2.05
1.02
1.94
1.640
1.610
1.357
1.350
1.331
1.321
1.313
1.310
1.298
1.293
f1 (ppm)

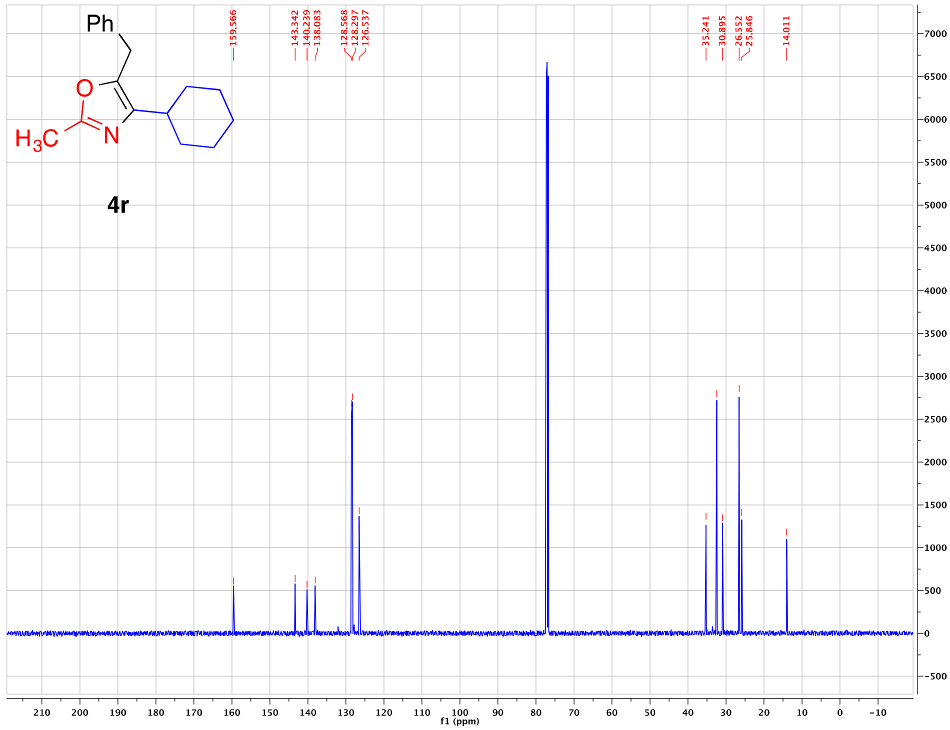

Ph
O
N
H3C
4r
159.566
143.342
140.239
138.083
128.568
128.297
126.537
35.241
30.895
26.552
25.846
14.011
f1 (ppm)
